# Supplementary material for: Sleep forms flexible context representations in toddlers
Source: Nat Commun. 2026 Oct 12;17:10699. doi: 10.1038/s41467-026-78303-4 (PMC13658235; doi:10.1038/s41467-026-78303-4)
Supplement: Supplementary file 3 [file 41467_2026_78303_MOESM3_ESM.pdf]

nature portfolio

Peer Review File

Sleep forms flexible context representations in toddlers

Corresponding Author: Professor Jan Born

This file contains all reviewer reports in order by version, followed by all author rebuttals in order by version.

Version 0:

Reviewer comments:

Reviewer #1

(Remarks to the Author)
This paper looks at how 2-3 year olds learn new contextual associations following an interval of sleep or wake. They find (1) following sleep, they were better able to learn new context memories relative to wake; (2) context memory formation was greater and non-context memory less following sleep and the opposite was true following wake; (3) these benefits of sleep were associated with sleep spindles and coupling of sleep spindles and slow oscillations.

At first I was very impressed how many significant results they found in a rather small sample of toddlers (that are known for providing noisy data). I still am impressed but I can't help but notice that many of these are rather small effects (p>.01) amongst what must be > 100 comparisons. I gather they looked at each electrode (x3), multiple measures (spindle density, amplitude, frequency, duration, SO density, amplitude, slope, duration, coupling (co-occurrence, vector length)), and these were multiplied by many measures of behavior (CMI, DMI, correct trials, context errors, random errors), etc. If, as I believe necessary, the significance is set at p<.01, there are many fewer significant effects. So I believe these results are over sold.

Next, I did not find the study motivation to align with the study that was carried out. Specifically, the goal was to examine the effects of sleep on spatial memory at an age when the hippocampus shows signs of immaturity (spatial and episodic memory starting to emerge). While the hippocampus does mature for a number of years, 25 months seems to be significantly beyond what is often thought to be the critical time frame of this development, 18-24 months (see Gomez & Edgin 2016). Moreover, they go on to do a task in which "encoding" (learning of object locations) and recall is all post- sleep/wake manipulation. They later describe the paradigm as a measure of "formation of abstracted context memory" and that it enhanced memory for the contexts. I can't tell, however, whether they are hypothesizing an effect of sleep on memory (the rooms learned before and recalled after sleep) or an effect of sleep on learning (encoding context-location associations before sleep and recalling after). They seem to use memory (and not learning or encoding) but it seems that was is being tested is actually sleep's effects on encoding. This might be interesting but does not seem aligned with the introduction. Moreover there are many analyses that seem ahypothetical (e.g., no predictions were made about behavior transitions, sleep physiology, etc). Additionally, they compare sleep intervals to wake intervals but my understanding is that those took place at different times of day (morning or afternoon as opposed to midday) and were different lengths (wake was a 90-min interval, sleep was 120-190 mins (90-160 mins sleep + 30 mins for sleep inertia). I.e., they are not necessarily capturing sleep v. wake effects but rather time-of-day effects and/or length of time effects.

Additionally, one approach to their analysis was to compute a contextual memory index and a decontextualized memory index and they suggest a dichotomy that contextual memory is increased by sleep and decontextual decreased by sleep. The problem is that CMI and DMI are not independent (they share the context errors component and must add up to 100). When one goes up (CMI) the other, by definition based on how it is calculated, has to go down. Take for instance:
Child A: 4 are correct, 0 is a context error, and 0 are random errors. (or 100%, 0%, and 0%). So, CMI =100/(100)=1 and DMI = 0/(0+0)=(technically undefined, but we'll call it 0)
Child B: 0 are correct, 0 context errors, 4 random errors. This would be 0%, 0%, and 100%. CMI=0/(0+0)= (technically undefined, but we'll call it 0) and DMI = 100/100=1.

There are also surprising effects of age. I am skeptical that age effects of the suggested magnitude would be evident with a small sample and a very very narrow age band. It would be important to show individual level data to be convincing.

The GNN analysis of behavior was surprising as it was not a motivated analysis and not explained well. The visual of Fig 3a was not helpful as it seems to show all probabilities are low other than that after the experimenter tells them to or engages

them, then they go look into the container. I'm not sure I get anything beyond that.

Finally, it is unclear that the event detection algorithms adopted were validated for use in data of this age. How do these compare to traditional spindle detection methods? Importantly, the values seem to contrast with other reports of this age group. For instance, the spindle density values range from about 1-5/min (Fig 4). McClain et al., report densities of .5-1.6 (2-3 years, C3/C4). Kwon et al. (2023), reports a mean of 2 spindles/min at this age. D'Arti et al (2018) report density 1.5-2 for this age range. Overall, a mean spindle density of ~3.5 is high for this age.

In summary, my evaluation is this: The conclusion (see line 320+) is that this is evidence that sleep-dependent consolidation uses the same hippocampus dependent mechanism in toddlers as adults. But (1) this is not a task that has a comparison in the adult literature, we don't know that it is necessarily hippocampal or benefited by sleep in adults; (2) showing a sleep benefit does not necessitate a hippocampal mechanism (see Gomez & Edgin's Childe Dev Perspectives paper for a nice discussion of this); and (3) it would not be too surprising to find this to be true as 26+ months is significantly beyond what is likely to be the critical hippocampal developmental period.

Minor:
- No description of how motivation was measured
- Wake was scheduled "depending on the activity levels of the child". What does that mean? This would not be replicable as described.
- "Children were animated to actively place the puppets…". What does this mean?
- Unclear how Cz is reference and data electrode – what was it re-referenced to? Could having C3 and C4 referenced to a different electrode than Cz explain the difference in outcomes across these electrodes?
- It is an exaggeration to call Fig 4a a robust correlation. The majority of the data falls outside the shaded area that I presume to indicate the confidence interval.
- Table S3 – says 'sindle'
-

(Remarks on code availability)

Reviewer #2

(Remarks to the Author)
This study tests whether a ~90-min nap after familiarization with two rooms helps toddlers aged 2-3 years form spatial context representations that guide later hide-and-seek retrieval. The authors found that children made fewer context-swap errors after sleep; spindle density and slow oscillation-spindle phase-amplitude coupling correlated with contextual memory. The paper addresses an important question in a vulnerable population using an elegant task, a within-subject crossover, rich behavioral annotation (including a graph-based analysis), and concurrent sleep EEG. The convergence of behavior and physiology is compelling. Statistics generally read as careful and robust. However, several design features (time-of-day confounds, retention-interval inequality, non-blinding) and some analysis choices (post-selection in the GNN) weaken the overall enthusiasm. Consequently, the manuscript should avoid causal claims ("sleep forms abstract context representations") and mechanistic interpretations ("adult-like hippocampal-neocortical dialogue"). Addressing these issues would markedly strengthen the paper.
Major concerns
• Sleep sessions were always midday; wake sessions occurred morning or afternoon. Thus arousal, prior wake time, and circadian phase differ by condition. If possible, I strongly suggest reporting exact session times and prior wake durations and including the time-of-day/prior wake as covariates. Furthermore, please elaborate on this issue in the discussion, outlining how a matched-time design might alter the interpretation.
• Naps could exceed 120 min and had an extra 30-min buffer; wake was ~90 min without a buffer. I would recommend considering the effective delay in analyses (e.g., include the delay as a covariate; provide a sensitivity analysis matched on delay).
• The experimenter likely knew the condition (nap vs. wake) and gave standardized prompts, scored behaviors, sometimes opened the correct container after 2.5 min. Furthermore, parents were passively present. At age 2-3, small variations in prompting and expectations can make a big difference. Please clarify blinding, standardization, and any checks for expectancy effects, and consider adding an analysis limiting to trials resolved without experimenter intervention.
• CMI = correct/(correct+context) and DMI = context/(context+random) are mathematically coupled. Could it be that the phrase "at the expense of decontextualized memory" reflects this coupling rather than a cognitive trade-off? Please elaborate on how the different measures relate to each other (what increases in one imply or do not imply for the others) in the context of task success (with larger values indicating better performance) or the opposite.
• The GNN classifies sleep vs. wake from encoding behavior and GNNExplainer identifies the "look-into-container - container-play" transition, which is then related to performance. This approach appears to be risking circular analysis, as the same data are used to identify features and then to test their relationship with performance. Please comment on this potential issue or include an additional analysis to mitigate it.
• You state "clear signs of hippocampal immaturity". Please specify what is meant (myelination, volume, connectivity, etc.) and summarize key findings. Please make explicit that no direct measures of hippocampal structure or function were collected here.
• With central scalp EEG only (C3/Cz/C4), correlations between spindles/SO-spindle coupling and behavior are consistent with thalamo-cortical involvement but do not establish hippocampal replay or an adult-like hippocampal–neocortical dialogue. I strongly recommend to tone down mechanistic equivalence claims and discuss alternatives (e.g., predominantly

thalamo-cortical consolidation at this age). The discussion appeals to the medial prefrontal cortex support for contextual abstraction, but this structure is also immature at 2-3 years. Please acknowledge this and temper the argument or cite evidence for early functional involvement despite immaturity.
Minor concerns
Introduction
• The opening paragraph presents SOs, spindles, and ripples in a rather condensed manner. Given the interdisciplinary audience, I recommend to briefly introduce each oscillation (frequency, generator, functional role) before discussing their coupling.
• The background on hippocampal maturation should be expanded: what developmental markers are relevant, and how they bear on consolidation.
• I suggest to clarify the scope and avoid promising an answer to regional relevance: the present design cannot disentangle hippocampal vs. cortical contributions.
Results
• The attenuation of sleep benefit with age is interesting. I suggest to further elaborate on this point and potential explanations.
• You define 10 behavioral states; please report how they were validated, inter-rater reliability, and whether rare states were pooled.
• In Figure 3, colors for experimenter vs parent are hard to distinguish.
• Please state explicitly whether all spindle analyses were conducted separately for slow (12–13 Hz) and fast (13–16 Hz) bands.
• In Figure 4, some scatter plots seem to have fewer points. I recommend adding the sample size for each analysis to every panel and clarify exclusions. I suggest to briefly define "surrogate data" in the caption.
Methods
• Please make explicit whether 28 is the final analyzed sample (after dropouts), how many were recruited initially and how the eight additional children excluded for insufficient cooperation/illness reconcile with the final sample size.
• Please specify whether EEG epochs with artifacts were rejected or interpolated, and the criteria for channel/epoch exclusion.
• Please state precisely the corrections for multiple comparisons used across analyses.
• "In models for correct trials and random errors the interaction was removed." – was this done because it yielded the most parsimonious model?
• Why is TST not included in Table S4?
• Does boldface in Tables S5 and S6 represent statistically significant results? Please specify and correct inconsistencies (e.g., Condition in S6 not bolded; Outcome×Condition in S5 bolded despite non-significance). I am confused by the numbers reported for the same analysis in the main text (page 7), as they do not match those presented in the table. If these values originate from a different analysis or model, please make this distinction clearer. Providing full model formulas throughout would be helpful.
• In Table S4, 2.94 ± 5.81 min, range 27–159.5 min appears inconsistent (that SD seems incompatible with the range). Please verify.

(Remarks on code availability)
The repository seems to contain only the code related to the GNN analysis. It would be helpful to see the remaining parts of the statistical analysis as well.

Version 1:

Reviewer comments:

Reviewer #2

(Remarks to the Author)
The authors have provided a comprehensive and thoughtful revision and have taken all comments seriously. I believe that the major concerns raised during the first review round have been adequately addressed.
I nevertheless have a few remaining concerns and suggestions:
1. In the abstract: "the sleeping toddler's brain seems to engage consolidation processes similar to those in adults"; given the acknowledged absence of direct hippocampal measures, this wording may still overstate mechanistic insights.
2. On page 3, the phrasing "including central output regions in the entorhinal cortex that develop rather late" could be more precise. "Rather late" is somewhat colloquial and vague.
2. While I appreciate the practical challenges of this population, several key analyses rely on relatively small subsamples (e.g., N = 22 for EEG analyses, N = 27 for transition probability analyses, and N = 17 for coupling analyses). I recommend to further acknowledge the implications of limited statistical power and stability of effect estimates in the Discussion.
3. Relatedly, the exclusion criterion for the coupling analysis would benefit from additional justification. The response states that "5 children were excluded … because only 17 out of the 22 children had a meaningful relationship between spindle amplitude and SO phase…". Please clarify more explicitly why this exclusion is methodologically necessary and whether this criterion was defined a priori.
4. Given the relatively small sample size, I wonder whether some of the mixed-effects models may be overparameterized. It would strengthen the manuscript to provide additional information on model performance and explained variance (e.g., marginal/conditional R^2 values where appropriate).
5. The response states that the number of independent statistical tests was "<15". For transparency, I suggest explicitly reporting the exact number in the manuscript itself.

6. While the Discussion has been substantially improved and appropriately toned down in many places, a few statements throughout the manuscript still appear somewhat stronger than warranted by the present data and could benefit from more cautious phrasing. For example:
• On page 17: “the toddlers in the sleep condition did not need to transit from opening to playing with the containers for successfully discriminating the spatial contexts”; this interpretation may overstate the conclusions that can be drawn from the exploratory GNN analyses and associated transition probabilities, particularly given the relatively modest classification accuracy of 65.79%.
• On page 19: “Introducing sleep after familiarization … allowed us to isolate the effects of sleep on pure context memory”; this seems stronger than justified given the authors’ later acknowledgment that effects on subsequent encoding processes cannot be fully excluded.
• “sleep helps form abstracted context representations” and related formulations referring to “generalized and abstract representations” or “flexible use in novel situations”; claims regarding abstraction/generalization may still go somewhat beyond the behavioral evidence provided.
• “persistent context memory” lasting >1.5 h; some formulations could benefit from greater conceptual precision in distinguishing persistence of contextual representations from persistence of the subsequently encoded object-location associations.
Overall, I think the manuscript would benefit from consistently maintaining the more cautious interpretational framing that the authors already adopted in some parts of the revision.

(Remarks on code availability)

Reviewer #3

(Remarks to the Author)
I am new to the review process, and read the reviews, the response and the revision. This paper is an exciting contribution to our understanding of early episodic memory. I have no further comments.

(Remarks on code availability)

Reviewer #4

(Remarks to the Author)
I co-reviewed this manuscript with one of the reviewers who provided the listed reports. This is part of the Nature Communications initiative to facilitate training in peer review and to provide appropriate recognition for Early Career Researchers who co-review manuscripts.

(Remarks on code availability)

Open Access This Peer Review File is licensed under a Creative Commons Attribution 4.0 International License, which

permits use, sharing, adaptation, distribution and reproduction in any medium or format, as long as you give appropriate credit to the original author(s) and the source, provide a link to the Creative Commons license, and indicate if changes were made.
In cases where reviewers are anonymous, credit should be given to 'Anonymous Referee' and the source.
The images or other third party material in this Peer Review File are included in the article's Creative Commons license, unless indicated otherwise in a credit line to the material. If material is not included in the article's Creative Commons license and your intended use is not permitted by statutory regulation or exceeds the permitted use, you will need to obtain permission directly from the copyright holder.
To view a copy of this license, visit https://creativecommons.org/licenses/by/4.0/

We thank the Reviewers for their insightful and constructive comments which we feel have greatly improved the quality and clarity of our manuscript. We have considered each point with great care and very much hope that we can satisfactorily address them with our arguments, additional analyses and extensive revisions that are presented in the following. All changes are highlighted in red in the revised manuscript.

REVIEWER 1

This paper looks at how 2-3 year olds learn new contextual associations following an interval of sleep or wake. They find (1) following sleep, they were better able to learn new context memories relative to wake; (2) context memory formation was greater and non-context memory less following sleep and the opposite was true following wake; (3) these benefits of sleep were associated with sleep spindles and coupling of sleep spindles and slow-oscillations.

Comment 1:

At first I was very impressed how many significant results they found in a rather small sample of toddlers (that are known for providing noisy data). I still am impressed but I can't help but notice that many of these are rather small effects (p>.01) amongst what must be > 100 comparisons. I gather they looked at each electrode (x3), multiple measures (spindle density, amplitude, frequency, duration, SO density, amplitude, slope, duration, coupling (co-occurrence, vector length)), and these were multiplied by many measures of behavior (CMI, DMI, correct trials, context errors, random errors), etc. If, as I believe necessary, the significance is set at p<.01, there are many fewer significant effects. So I believe these results are over sold.

Response 1:

We appreciate the reviewer's concern regarding sample size, effect magnitude, and multiple testing, and we agree that these issues are particularly important in toddler research where data can be noisy.

First, we would like to clarify that effect size cannot be inferred from the p-value alone. A threshold such as p < .01 versus p < .05 does not by itself distinguish "small" from "large" effects, but rather reflects a stricter Type-I error criterion. To address the Reviewer's concern more directly, we now report effect sizes for all main tests (both behavioral and EEG–behavior relations) in the Results, which allows readers to judge the magnitude and precision of the effects beyond the p-values. In the sleep and developmental literature, small-to-medium effects have been shown to be meaningful. Indeed, the effects sizes for our main effects are in the medium range.

Second, the number of independent statistical tests is substantially lower (< 15) than the reviewer's rough estimate of "> 100 comparisons". Our main analyses were all hypothesis-driven and model-based, not a collection of many separate univariate tests. Specifically:

- For the key behavioral questions (percent correct, context errors, random errors; CMI/DMI), we used a small set of linear mixed-effects models that included all predictors of interest and covariates in the one and the same single model, rather than

running multiple separate tests for each factor and outcome (please, see Tables S5–S6 for the full model structure).

- For EEG–behavior relations, we did not test every parameter at every electrode against every behavioral outcome separately but included the parameters in one moel (see Table S7).
- Our hypothesis-driven spindle-SO coupling analyses focused on Cz, where coupling was actually observed, rather than treating all three electrodes as independent discovery sites.

Thus, the notion that we performed on the order of 100 independent comparisons is incorrect and does not accurately reflect our analytic strategy.

Third, where multiple testing is a real concern, we took explicit measures:

- In the mixed-effects models post-hoc contrasts were corrected for multiple comparisons (Holm-Bonferroni, as specified in the Statistical Analysis section p. 34).
- For the exploratory correlations between sleep macro-architecture and CMI (Table S4), which we labeled as exploratory also in the results section.
- The graph neural network analysis, which we specified as hypothesis-generating and interpret with caution (see details in the response to Comment 4).

We therefore believe that the results are not "oversold", but that our statistical reporting may not have been sufficiently clear in the original version of the manuscript. We now explicitly distinguish between confirmatory, hypothesis-driven analyses and clearly labeled exploratory analyses. Moreover, on several occasions in the Results section, we now reference to the type of statistical approach used.

We also understand that the hypotheses could have been stated more clearly and have, thus, modified the final paragraph of the **Introduction** (p. 5, ll. 65 – 75):

"We first hypothesized that, compared to an equivalent period of wakefulness, a post-familiarization nap would selectively improve contextual aspects of memory, expressed as fewer context errors and a higher Contextualized Memory Index (CMI), without necessarily changing overall accuracy or random errors. Second, based on prior work linking sleep spindles to memory consolidation in children (Bastian et al., 2023), we expected that higher spindle density during NonREM sleep would be associated with a lower context error rate and a higher CMI, indicating more robust binding of object information to its spatial context. Third, we hypothesized that stronger coupling between slow oscillations and spindles would further predict decreases in context errors and increases in CMI, consistent with the view that precisely timed SO–spindle coordination supports the consolidation of abstracted context representations in early childhood."

Comment 2a:

Next, I did not find the study motivation to align with the study that was carried out. Specifically, the goal was to examine the effects of sleep on spatial memory at an age when the hippocampus shows signs of immaturity (spatial and episodic memory starting to emerge). While the hippocampus does mature for a number of years, 25 months seems to be significantly beyond what is often thought to be the critical time frame of this development, 18-24 months (see Gomez & Edgin 2016).

Response 2a:

We appreciate the Reviewer's reference to Gómez & Edgin (2016) and agree that this work is central for characterizing the early trajectory of hippocampal development and memory. However, more recent developmental models emphasize a protracted, functionally heterogeneous maturation of hippocampal-dependent memory, in which different relational binding capacities emerge sequentially across early childhood rather than simultaneously (see Bevandic et al., 2024). Crucially, the age range tested here (25–34 months) corresponds to a transitional phase in which toddlers can begin to form allocentric representations and flexible what–where associations across distinct spatial contexts, but do so fragilely and inconsistently, particularly under wake conditions. This is precisely the developmental window in which sleep-dependent consolidation effects may be most informative and is supported by our Age x Condition interaction effect on context error rate (**Fig. 2d**).

Even Gómez & Edgin do not restrict hippocampal maturation to a narrow window ending at 24 months, but emphasize an extended developmental trajectory from infancy into the preschool years, with "substantial continued hippocampal development between 2 and 5 years" (p. 59). These developments parallel improvements in episodic and spatial memory functions as demonstrated by structural and functional MRI work, and behavioral studies (Keresztes et al., 2017; Riggins et al., 2016; Blankship et al., 2017; DeMaster et al., 2014; Ribordy et al., 2013) showing that context and relational memory to strengthen well into the school period.

Nevertheless, we want to acknowledge that the age before 24 months is very interesting in this regard, and obviously our findings cannot directly address how sleep impacts context memory during the earliest phases of hippocampal development. We therefore toned down the respective sections in the Introduction and Discussion. We also point out more clearly in the Discussion that future work may adapt our paradigm to this younger age range (< 24 months). This is indeed a direction we are currently pursuing in our lab.

Introduction (p.3, ll.14-21; p.4, ll.35-40):

"Indeed, structural and functional hippocampal development follows a prolonged trajectory[23], involving processes such as myelination of neuronal axons and ongoing neurogenesis, which continue into later childhood or even adulthood [24–27]. Specifically, subregions of the hippocampus involved in the tri-synaptic pathway suggested to be crucial for forming an episodic memory engram[23], namely CA3 and dentate gyrus, develop well into the second year at least in rodents[28,29]. Recent volumetric measurements in humans corroborate these findings showing growth of CA3 and dentate gyrus until early adulthood[30]."

"Although behavioral evidence suggests an inflection point for episodic memory formation in the second year of life [41], these memories are still fragile and easily disrupted well into

preschool age [39,42]. We therefore focused on toddlers aged 2–3 years, an age at which spatial context representations are present but not yet adult-like, to ask whether sleep strengthens the consolidation and abstraction of such representations during this early phase of hippocampal development."

Discussion (p. 23, ll. 465-476):

"It should be noted that our sample comprised toddlers aged 2–3 years, i.e., slightly beyond the 18–24-month period that has been proposed as particularly critical for the onset of hippocampal-dependent memory. While converging evidence indicates substantial continued hippocampal and episodic memory development between 2 and 5 years, our findings therefore speak specifically to an early toddler stage, in which context memory is present but still immature, rather than to the very first emergence of hippocampal-dependent memory. Whether similar sleep-related benefits for contextual memory and similar thalamo-cortical mechanisms can already be observed in younger infants (< 25 months) remains an open question. Future work adapting the current paradigm to earlier developmental stages will be essential to determine how early in life the sleep-dependent consolidation of spatial context representations emerges and how it changes across the extended trajectory of hippocampal maturation."

Comment 2b:

Moreover, they go on to do a task in which "encoding" (learning of object locations) and recall is all post- sleep/wake manipulation. They later describe the paradigm as a measure of "formation of abstracted context memory" and that it enhanced memory for the contexts. I can't tell, however, whether they are hypothesizing an effect of sleep on memory (the rooms learned before and recalled after sleep) or an effect of sleep on learning (encoding context-location associations before sleep and recalling after). They seem to use memory (and not learning or encoding) but it seems that was is being tested is actually sleep's effects on encoding. This might be interesting but does not seem aligned with the introduction.

Response 2b:

Thank you very much for raising this point. We agree that distinguishing sleep effects on context memory from sleep effects on encoding of object–location associations is conceptually important. While dissociating these kinds of effects through appropriate control conditions is very difficult to achieve in the framework of the present paradigm, we believe that a simple effect of sleep on encoding the object-location associations is highly unlikely and can, to the furthest extend, be ruled out through appropriate control analyses.

To address this comment, we first carefully considered alternative control designs:

1. Sleep vs. wake groups performing the hide-and-seek task without prior context familiarization. However, this would fundamentally change what the task measures. In such a design, performance would mainly reflect on-line adaptation to a novel environment across trials, not the use of pre-existing context representations. Especially in toddlers, novelty effects in the first trials would likely dominate behavior,

with children primarily engaged in exploring the context-relevant objects rather than focusing on the memory demands of the task. This would no longer be comparable to our current conditions.

2. A sleep control group familiarized with only one context (e.g., room A but not B). The idea would then be to test whether children make fewer context errors in the familiarized room A than in the unfamiliar room B. However, such a contrast would also be difficult to interpret. Any advantage for room A could again reflect reduced novelty and distraction rather than sleep-dependent consolidation of context memory. In other words, differences between A and B would be confounded with differences in novelty and attentional demands rather than isolating an effect of sleep on contextual representations.

Thus, including a context familiarization phase is essential for this paradigm and aligns with the original design choices by Newcombe et al. (2014).

Considering the limitations of the possible control designs, we instead exploited variation in children's behavior during the familiarization phase itself. Specifically, we coded a state event termed "targeted play", which we used as a behavioral marker of context familiarization (following Tamis-LeMonda & Masek, 2023). This category captures the time the child actively engaged in playing with context-relevant items during familiarization (i.e. objects unique to that room), explicitly excluding items that were identical across contexts (containers, furniture, door handles, etc.). In addition, we included the transition probability from "Looking into container" to "Container play" during encoding as a confound predictor, to control for the effects of sleep on encoding behavior.

We found a significant interaction between Targeted Play (in % of trial duration) during familiarization and Sleep/Wake on context error rate ($\beta = 0.523$, SE = 0.20, $P = 0.018$, 95% CI [0.10, 0.95]).Follow-up analyses showed that more targeted play during familiarization predicted fewer context errors in the sleep condition (sleep: $\beta = -0.297$, SE = 0.14, $P = 0.048$, 95% CI [−0.59, −0.01]), whereas targeted play had no effect in the wake condition (wake: $\beta = 0.228$, SE = 0.15, $P = 0.139$, 95% CI [−0.08, 0.54]). Thus, greater engagement with the context-relevant features only benefitted later context memory when the interval between familiarization and retrieval was filled with sleep, which is difficult to explain by a pure "sleep improves encoding" account.

Furthermore, if there were simply effects of sleep on alertness or task engagement at encoding one would expect altered motivation during encoding between conditions. Behavioral motivation scores shown in **Fig. S1** were adapted from Berhenke et al. (2011) incorporating mastery motivation behaviors (persistence and competence), strategy use (help-seeking), and emotion expression (positive affect, interest/arousal, sadness, confusion, anxiety, anger, frustration, hostility, and shame). However, in **Fig. S1** we demonstrated no difference in motivation between conditions. As an additional control analysis, we show that motivation during encoding was not related to the context error rate across conditions (sleep: $r = -0.054$, $P = 0.788$; wake: $r = -0.043$, $P = 0.830$).

We have added these analyses to the Methods and Results sections and included a new supplementary figure (**Fig. S2**):

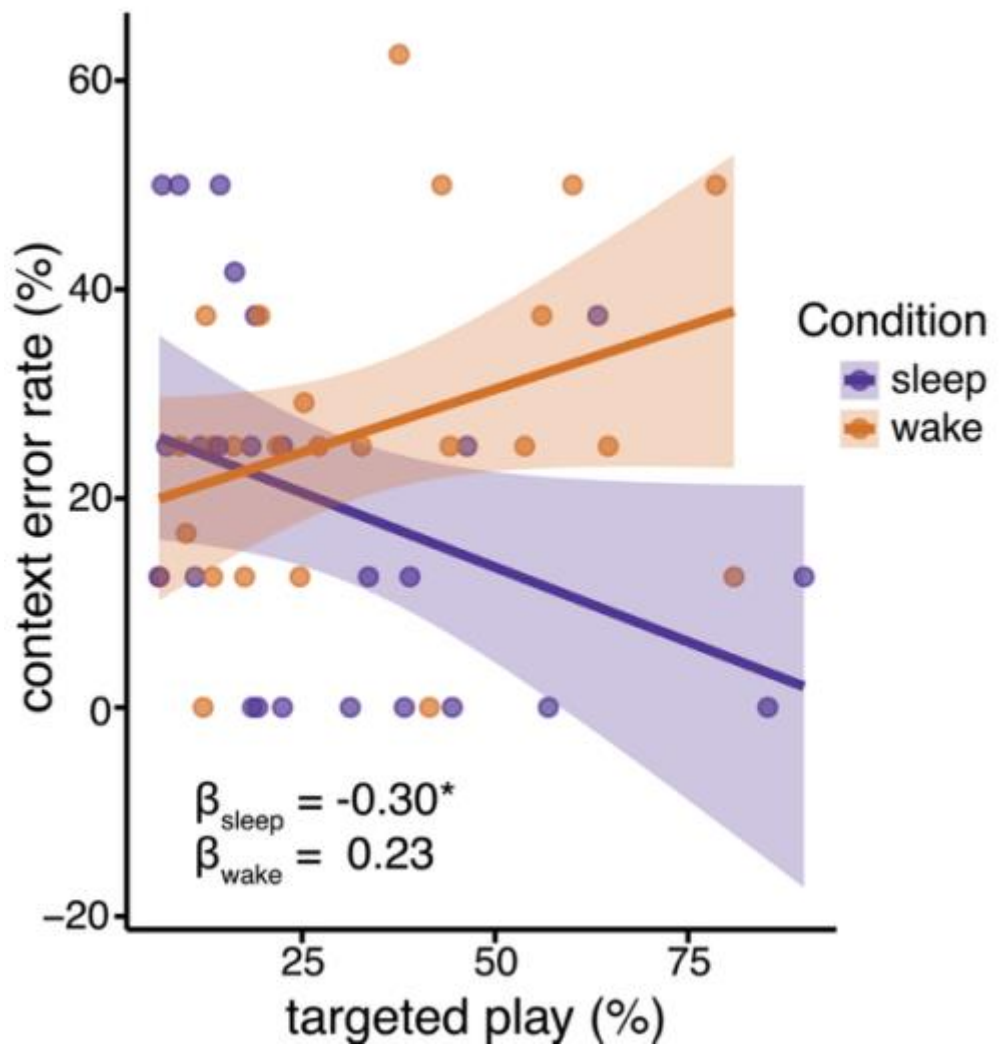

Figure S2. Distinct effects of context familiarization on context error rate after sleep and wakefulness. *Mean (±SEM) for the effect of targeted play during context familiarization (i.e. playing with context relevant items; see Table S1) on context error rate for the sleep (purple) and wake (orange) condition. Engaging in more targeted play (relative to the trial duration) during context familiarization was associated with fewer context errors after sleep (P = 0.048) and no effect after wakefulness (P = 0.139). Individual data points are overlayed.*

Methods (p. 29f, ll.618-633):

"Among the target behaviors of the familiarization phase, the rater coded a state event termed "targeted play" as a marker of context familiarization[73]. Targeted play was defined as periods during which the child actively engaged with objects that were unique to the specific room (e.g. zoo animals, doctor's kit, kitchen utensils or train tracks), excluding items that were identical across contexts (containers, furniture, door handles, etc.). For the familiarization in each room, we computed the percentage of time spent in targeted play relative to the total familiarization duration. This measure was used to test the relevance of context familiarization for the two conditions. The rater also coded any verbal or non-verbal actions by the experimenter or parent that could potentially cue the child. Trials containing non-allowed prompting were excluded from analysis.

Motivation during encoding of the hide-and-seek task was rated from the video recordings on a 0–10 scale adapted from [74], […]."

Methods (p. 35, ll.787-793):

"To test whether context familiarization contributed to subsequent memory performance independently of sleep-related effects on encoding, we conducted an LMM analysis on behavior during the familiarization phase. Context error rate served as the dependent variable and was modeled as a function of the percentage of targeted play during familiarization, experimental condition (sleep vs. wake), their interaction, controlling for the transition probability between the critical behavioral states at encoding (0–1). Participant identity was included as a random intercept."

Results (p. 9, ll. 134-146):

*"Finally, to establish a direct link between context familiarization and later context memory, we examined in an exploratory analysis whether children's engagement with context-relevant objects during familiarization predicted context error rates at retrieval. The proportion of time spent in targeted play (i.e. playing with context-specific items; see Table S1) relative to trial duration differentially predicted context error rate after sleep and wakefulness (**Fig. S2**; Condition × Targeted Play: β = 0.523, SE = 0.20, P = 0.018, 95% CI [0.10, 0.95]). Follow-up analyses showed that more targeted play was associated with fewer context errors after sleep (β = −0.297, SE = 0.14, P = 0.048, 95% CI [−0.59, −0.01]) but had no effect after wakefulness (β = 0.228, SE = 0.15, P = 0.139, 95% CI [−0.08, 0.54]). Thus, richer interaction with the contextual features during familiarization benefitted context memory specifically when followed by sleep. Furthermore, sleep likely did not have unspecific effects on attention and task engagement as motivation during encoding was not related to the context error rate across conditions (sleep: r = -0.054, P = 0.788; wake: r = -0.043, P = 0.830)."*

Nevertheless, even though our control analyses strongly support the view of sleep-dependent formation of a context representation, we agree with the Reviewer that we should elaborate on the rationale of our design in the manuscript.

Discussion (p. 21, ll. 421-439):

"Despite the advancement in understanding sleep effects on spatial context memory formation, several limitations of the present study should be acknowledged. One major limitation concerns the interpretation of our findings with respect to sleep effects on representations of the contexts experienced during the familiarization phase versus effect on encoding of the hide-and-seek task. In our paradigm, the familiarization phase establishing contexts preceded the sleep/wake manipulation, whereas the encoding of object–location associations and their retrieval in the hide-and-seek task occurred thereafter. Control analyses revealed a sleep-specific relation between behavioral indicators of familiarization strength (targeted play) and later context errors while controlling for behavioral sequences during encoding affected by sleep (i.e. looking into container → container play). Together with the absence of condition differences in motivation at encoding, these findings are consistent with the view that sleep strengthened an underlying representation of the spatial contexts independent of its effects on encoding processes. Indeed, non-specific effects of sleep, e.g., increasing alertness are highly unlikely to express themselves in a specific enhancement of context memory but would behave been expected to similarly enhance object memory (i.e., to decrease random errors. At the same time, we cannot fully exclude that sleep also modulated subsequent encoding processes. A problem that is basically difficult to resolve by appropriate control conditions in the context of the present task paradigm and may be addressed in future work."

Comment 2c:

Moreover there are many analyses that seem ahypothetical (e.g., no predictions were made about behavior transitions, sleep physiology, etc).

Response 2c:

There may have been some confusion regarding our hypotheses. We have now clarified that our main analyses were all driven by a priori hypotheses (please, see our response to Comment 1), except the GNN analysis which was hypothesis-generating (see our response to Comment 4).

Comment 2d:

Additionally, they compare sleep intervals to wake intervals but my understanding is that those took place at different times of day (morning or afternoon as opposed to midday) and were different lengths (wake was a 90-min interval, sleep was 120-190 mins (90-160 mins sleep + 30 mins for sleep inertia). I.e., they are not necessarily capturing sleep vs. wake effects but rather time-of-day effects and/or length of time effects.

Response 2d:

This is an important point and largely overlaps with a concern by Reviewer 2. Thus, we would like to direct you to our answer to Comment 1 (time-of-day effects) and Comment 2 (retention interval duration) of Reviewer 2. In brief, we did not find confounding effects of time-of-day and retention interval for context error rate or CMI/DMI. In addition, neither of the variables accounted for the variance explained by the Sleep/Wake manipulation. We included these control analyses in the Supplementary Materials.

Comment 2e:

Additionally, one approach to their analysis was to compute a contextual memory index and a decontextualized memory index and they suggest a dichotomy that contextual memory is increased by sleep and decontextual decreased by sleep. The problem is that CMI and DMI are not independent (they share the context errors component and must add up to 100). When one goes up (CMI) the other, by definition based on how it is calculated, has to go down. Take for instance:

Child A: 4 are correct, 0 is a context error, and 0 are random errors. (or 100%, 0%, and 0%). So, CMI =100/(100)=1 and DMI = 0/(0+0)=(technically undefined, but we'll call it 0) Child B: 0 are correct, 0 context errors, 4 random errors. This would be 0%, 0%, and 100%. CMI=0/(0+0)= (technically undefined, but we'll call it 0) and DMI = 100/100=1.

Response 2e:

Yes, CMI and DMI are obviously mathematically coupled because they share the context error component but they do not necessarily add up to 100%, meaning that, in statistical terms, while both parameters share variance, they also have independent variance, and our statistical testing focused on this independent variance. We address this comment in detail with an example in our response to Comment 4 by Reviewer 2. In brief, we used a multivariate linear mixed-effects model in which both indices were included as repeated outcome measures within each participant. Importantly, this model structure does not assume that CMI and DMI are entirely independent constructs allowing us to interpret significant effects of Sleep/Wake

on CMI and DMI as they pertain to the non-correlated, i.e., uncoupled variance of these parameters.

Comment 3:

There are also surprising effects of age. I am skeptical that age effects of the suggested magnitude would be evident with a small sample and a very very narrow age band. It would be important to show individual level data to be convincing.

Response 3:

We were also somewhat surprised by the age effect given the relatively narrow age range in our sample. However, based on Newcombe et al. (2014), who reported clear age-related differences in performance, we expected this task to be sensitive to developmental variation. This is why age was included as a covariate.

To examine the effect of age more finely, we re-expressed age in days (time from birth to the first test session) instead of months. With this more precise measure, the Sleep/Wake × Age interaction remained significant. We adjusted **Fig.2, Tables S5/S6** and the relevant statistics throughout the Results section (marked in red).

In addition, we corrected an omission of individual data points for the age effects in **Fig. 2d,f.** This was a mistake.

We now also elaborate on the interpretation and limitations of the age effects in the **Discussion** (p. 23f, ll.477-487):

"A notable aspect of our findings is the modulation of sleep effects by age within a relatively narrow 25-34-month range. Although our two-room paradigm is known to be sensitive to age-related differences in context memory [38], our restricted age range warrant a cautious interpretation of these effects. To capture the narrow age range more precisely, we expressed age in days rather than months. Although the modulating effect of Age remained, significant when age was expressed in a more fine-grained way, in days rather than months, it was not significant in follow-up comparisons and individual datapoints showed large variability. We therefore view the present age effects as suggestive rather than definitive, consistent with the idea that sleep-related benefits for context memory may be particularly pronounced at the younger end of the toddler period, but in need of replication in larger samples and with broader age ranges."

Comment 4:

The GNN analysis of behavior was surprising as it was not a motivated analysis and not explained well. The visual of Fig 3a was not helpful as it seems to show all probabilities are low other than that after the experimenter tells them to or engages them, then they go look into the container. I'm not sure I get anything beyond that.

Response 4:

We appreciate the Reviewer's concerns about the motivation and clarity of the graph neural network (GNN) analysis. We agree that, in its original form, the description of this analysis and **Fig. 3a** did not sufficiently convey why this approach is informative beyond the obvious fact that children often look into the container after the experimenter prompts them are they actively engage in the task.

Conceptually, the goal of the GNN analysis was not to re-discover such trivial transitions, but to ask whether the full temporal structure of children's encoding behavior in interaction with the other actors in the room contains information that distinguishes sleep and wake conditions, and, importantly, to identify which specific transitions contribute most strongly to this distinction. This analysis was indeed hypothesis-generating and not confirmatory. It follows a growing literature using sequence- and network-based methods to extract structure from rich behavioral time series, rather than reducing behavior to a small set of averages (e.g., Abney et al., 2018; Xu et al., 2020). Graph neural networks appear to be well suited to this purpose because they can learn from graph-structured data while preserving information about both nodes (behaviors) and edges (transition probabilities).

The key result was that the transition from "look into container" → "container play" emerged as a discriminative feature for sleep vs. wake, i.e., a pattern of child-initiated exploration of the container which seemed to be more relevant for the wake rather than the sleep group for successfully learn the correct puppet location (see **Fig. 3**). In other words, when children slept after context familiarization, they appeared to need less sensorimotor engagement with the containers during encoding to remember the locations correctly, consistent with the idea that they are relying more on an already-formed context representation of the room as a reference frame. In the wake condition, in contrast, children seem to compensate for weaker context representations by more intensively exploring the containers, suggesting a greater reliance on object-/container-based encoding rather than on an abstracted context map.

Nevertheless, given the exploratory nature of this approach, we have further toned down this part in the Results section, and importantly, to clarify our GNN analysis, we have added the motivation to the Methods section, deleted panel 3a and instead expanded **Fig. S4**. For readers interested in the implementation, the code is already available in our public repository (as stated in the Code Availability section).

Methods (p. 31f, ll. 673-687):

"The video-coded behavior during familiarization and encoding yields rich temporal sequences of discrete states (e.g., looking into a container, playing with context-relevant objects, seeking help from the parent or experimenter). Rather than reducing these sequences to a few summary counts or durations, we modeled them as behavioral graphs, in which nodes represent behaviors and directed, weighted edges represent transition probabilities between behaviors within a trial. This graph-based representation preserves information about both which behaviors occur and how they are chained over time. We then used a graph neural network (GNN) as an exploratory, hypothesis-generating tool to ask (i) whether the structure of encoding behavior contains information that discriminates sleep from wake trials and (ii) which specific behavioral transitions are most informative for that discrimination and for subsequent memory performance. In this way, the GNN analysis complements our conventional behavioral measures by capturing the organization of children's interactions with

the context and containers, rather than only their overall frequency or duration, and provides candidate behavioral signatures of how post-familiarization sleep may alter encoding strategies."

Figure S4:

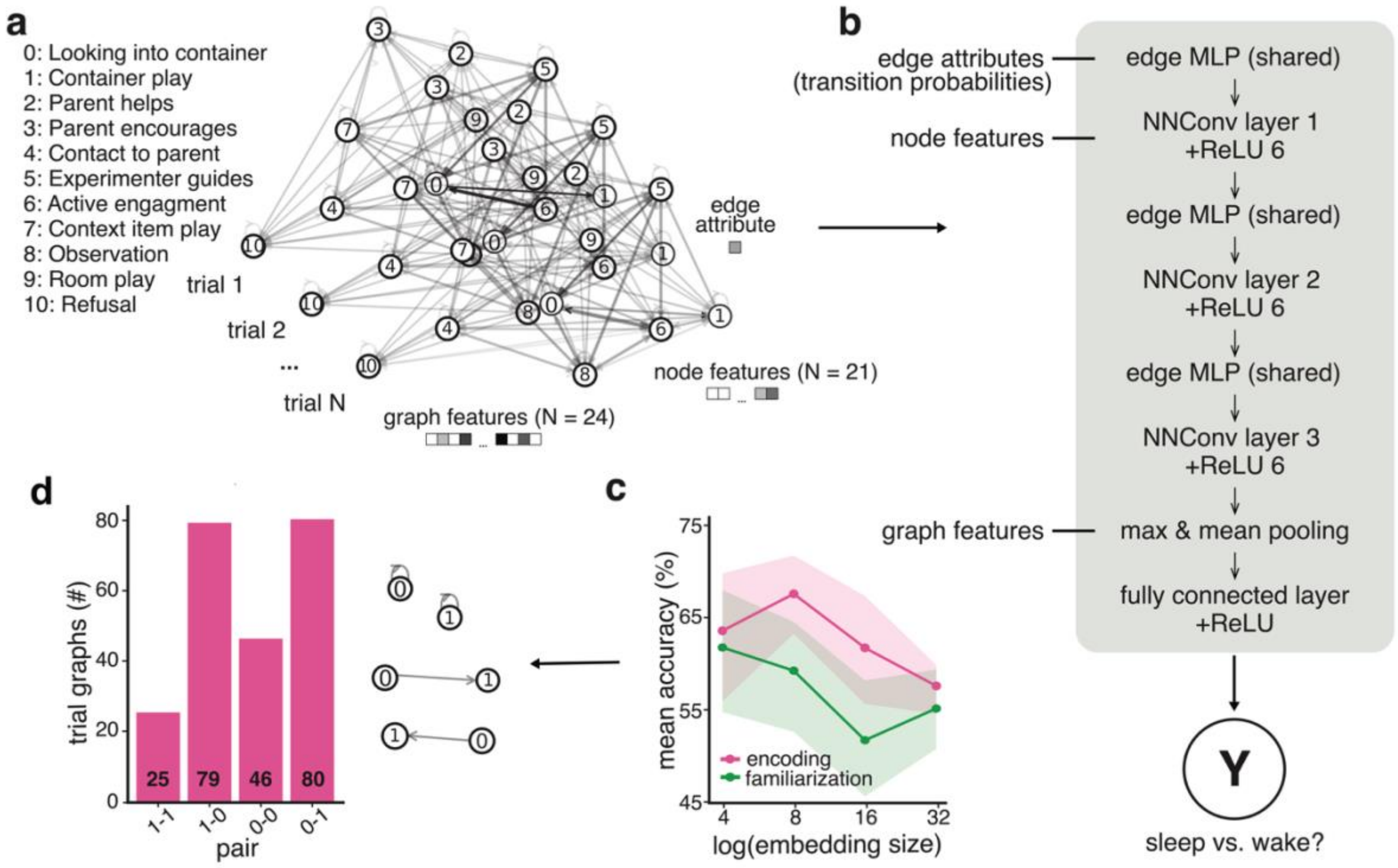

***Figure S4. Graph neural network analysis, embedding size comparison and node pair relevance. a,** Trial-wise directed encoding graphs constructed from video-scored behavioral sequences. Nodes represent distinct behavioral states (labels 0–10), edges indicate transition probabilities between behaviors, and node- and graph-level features encode behavioral, task-related, and demographic information. One graph was generated per trial. **b,** Graph neural network (GNN) architecture used for classification. Node features were processed by three edge-conditioned convolutional layers (NNConv) in which edge attributes (transition probabilities) were transformed into convolutional weight matrices via a shared multilayer perceptron (MLP). ReLU6 activations were applied after each convolution. Node embeddings were aggregated using global mean and max pooling and concatenated with graph-level features before classification by a fully connected layer with ReLU activation, yielding a binary prediction (sleep vs. wake). **c,** Mean (±SEM) 10-fold cross-validation (CV) accuracies across embedding sizes (i.e. the dimensionality of the latent graph representations) of the GNN at familiarization (green) and encoding (pink). The embedding size with the highest CV-accuracy (familiarization: 4, encoding: 8) was used for the final test. **d,** Histograms of the top 20% node pairs (illustrated on the right) in the graphs of each trial used for the GNN predictions at encoding. The node pair most often identified to differentiate between sleep and wake conditions was 0-1.*

Results (p. 11, ll. 176 & 182f):

“In an exploratory fashion, we examined whether sleep affected the child’s behavior already at encoding (hiding the puppets) and whether such influence could predict the formation of spatial context memory.”

*“**Fig. S4a** shows exemplary trial graphs.”*

Comment 5:

Finally, it is unclear that the event detection algorithms adopted were validated for use in data of this age. How do these compare to traditional spindle detection methods? Importantly, the values seem to contrast with other reports of this age group. For instance, the spindle density values range from about 1-5/min (Fig 4). McClain et al., report densities of .5-1.6 (2-3 years, C3/C4). Kwon et al. (2023), reports a mean of 2 spindles/min at this age. D’Arti et al (2018) report density 1.5-2 for this age range. Overall, a mean spindle density of ~3.5 is high for this age.

Response 5:

The spindle detection used here is rather traditional, and was first published in 2002 by Moelle and colleagues. Since then, this algorithm has been applied in different species and was further taylored to human sleep EEG (see https://github.com/Frederik-D-Weber/sleeptrip). The spindle detection algorithm has also been validated in several independent samples of children of a similar age (Kurz et al., 2024) and even younger infants (e.g., Friedrich et al., 2019; Friedrich et al., 2022). For example, Kurz et al. (2024) reported an average spindle density of 3.31 spindles/min over central electrodes in toddlers, which is highly consistent with the mean density of 3.37 spindles/min observed in our sample.

During the analysis, we visually inspected the detected events at the single-subject level. To illustrate that the algorithm captures physiologically plausible spindles, we here show an average spindle waveform and random single events (30-s segment) from a randomly selected participant:

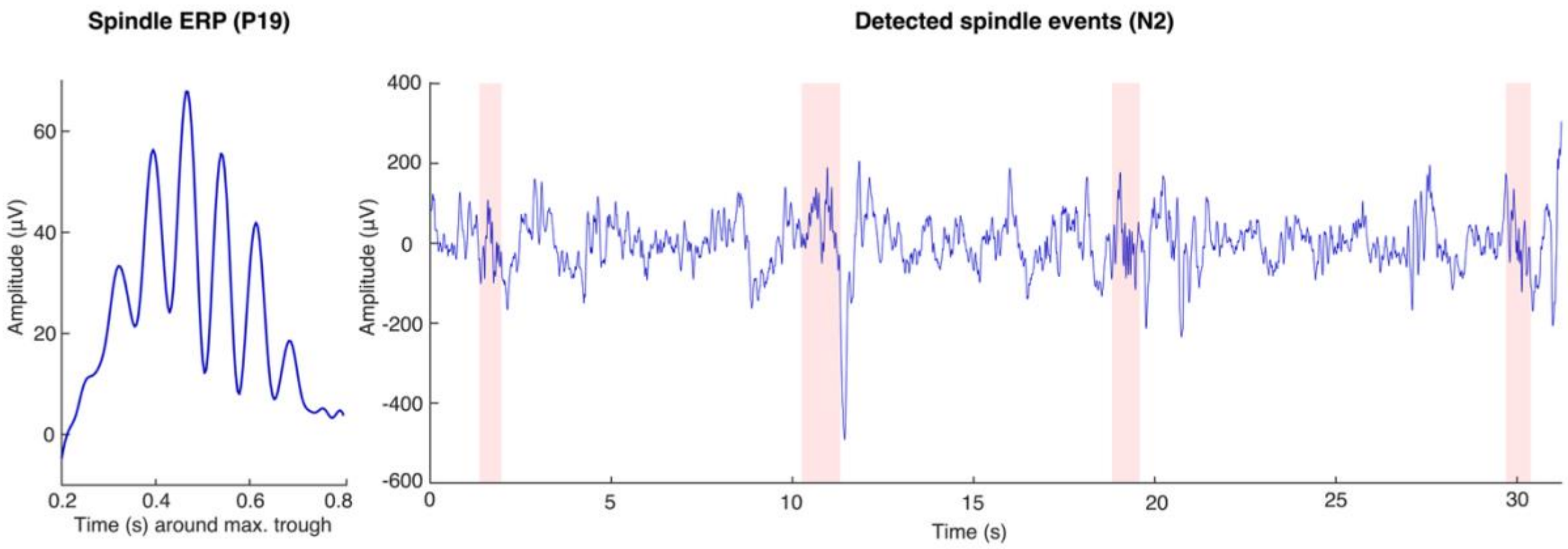

REVIEWER 2

This study tests whether a ~90-min nap after familiarization with two rooms helps toddlers aged 2-3 years form spatial context representations that guide later hide-and-seek retrieval. The authors found that children made fewer context-swap errors after sleep; spindle density and slow oscillation-spindle phase-amplitude coupling correlated with contextual memory. The paper addresses an important question in a vulnerable population using an elegant task, a within-subject crossover, rich behavioral annotation (including a graph-based analysis), and concurrent sleep EEG. The convergence of behavior and physiology is compelling. Statistics generally read as careful and robust. However, several design features (time-of-day confounds, retention-interval inequality, non-blinding) and some analysis choices (post-selection in the GNN) weaken the overall enthusiasm. Consequently, the manuscript should avoid causal claims ("sleep forms abstract context representations") and mechanistic interpretations ("adult-like hippocampal-neocortical dialogue"). Addressing these issues would markedly strengthen the paper.

Comment 1:

Sleep sessions were always midday; wake sessions occurred morning or afternoon. Thus arousal, prior wake time, and circadian phase differ by condition. If possible, I strongly suggest reporting exact session times and prior wake durations and including the time-of-day/prior wake as covariates. Furthermore, please elaborate on this issue in the discussion, outlining how a matched-time design might alter the interpretation.

Response 1:

Thank you for raising this important point regarding potential circadian and arousal-related confounds. We agree that differences in time-of-day and prior wake duration represent a limitation of our design and have now addressed this issue both analytically and in the Discussion.

The scheduling of sessions was guided by ethical and practical considerations, as we aimed to avoid sleep deprivation or excessive fatigue in toddlers. Accordingly, all sleep sessions took place during the children's habitual midday nap, whereas wake sessions were scheduled either in the morning or in the afternoon, depending on the child's individual chronotype (morningness vs. eveningness; see Randler Faßl & Kalb, 2017). Importantly, wake sessions were arranged such that the time since the last sleep episode (night sleep or habitual nap) was comparable between morning and afternoon groups.

To address the Reviewer's concern, we have now conducted additional control analyses. First, we included prior wake duration as a covariate in the linear mixed-effects models predicting context error rate as well as the CMI/DMI. Prior wake duration did not significantly predict any memory outcome, nor did it interact with condition (all $P > 0.549$). Second, within the wake condition, we directly compared children tested in the morning ($n = 15$) versus afternoon ($n = 13$). No differences were observed between these subgroups for context error rate ($P = 0.765$).

These additional analyses have been added to the Supplementary Information. Furthermore, we have expanded the Discussion to explicitly address the limitation:

Methods (p. 35, ll. 768f):

"In additional control analyses, Retention Interval (in min) and prior Wake Duration (in min) were included as confound predictors."

Supplementary Methods (p. 4, ll. 73f):

"To control for time-of-day effects, an independent samples t-tests was performed to compare context error rates for wake participants tested in the morning versus afternoon."

Supplementary Note 1 (p. 5, ll. 117-128):

"To assess whether differences in prior wake duration or time-of-day contributed to the observed memory effects, we performed additional control analyses. Prior wake duration before familiarization was entered as a covariate into the linear mixed-effects models for context error rate, CMI, and DMI. Prior wake duration neither showed a main effect on context error rate ($\beta = 0.06$, SE = 0.06, $P = 0.315$, 95% CI [-0.05 0.17]), or CMI/DMI ($\beta < -0.001$, SE = 0.001, $P = 0.305$, 95% CI [-0.002 0.001]), nor did it interact with the Sleep/Wake condition in any model (all Sleep/Wake x Wake Duration interaction $P > 0.435$). In addition, memory performance within the wake condition did not differ between children tested in the morning ($n = 15$) versus the afternoon ($n = 13$). Independent-samples comparisons revealed no significant differences for context error rate ($t_{22} = 0.31$, $P = 0.765$, 95% CI [-10.89 14.61], $d = 0.13$). Together, these do not provide any hints that the observed sleep-related differences in contextual memory is due to differences in prior wake duration or time-of-day effects."

Discussion (p. 22, ll. 440-452):

"Additionally, sleep and wake conditions were not fully matched for time-of-day. While all sleep sessions occurred during the toddlers' habitual midday nap, wake sessions took place either in the morning or in the afternoon according to the children's chronotype. Consequently, circadian phase and time-of-day–dependent fluctuations in arousal cannot be entirely ruled out as contributing factors. However, several observations argue against a primary role of such effects. First, prior wake duration did not predict any of the memory measures when included as a covariate, nor did it interact with the Sleep/Wake condition. Second, within the wake condition, memory performance did not differ between children tested in the morning versus the afternoon. Finally, the observed sleep effects were selective to measures of contextual memory, whereas overall accuracy and random error rates were unaffected, which would be unexpected if general alertness or fatigue were driving the results. Although a fully time-matched design would be preferrable to rule out circadian effects, it is not possible without sleep depriving habitually napping children, leading to confounding effects of fatigue during the memory task."

Comment 2:

Naps could exceed 120 min and had an extra 30-min buffer; wake was ~90 min without a buffer. I would recommend considering the effective delay in analyses (e.g., include the delay as a covariate; provide a sensitivity analysis matched on delay).

Response 2:

Thank you for this helpful comment. We agree that differences in the effective delay between familiarization and memory testing could influence performance and therefore warrant careful consideration.

The total break time for the sleep group (mean = 159.42 min) tended to be longer than for the wake group (mean = 134.63 min). However, we aimed at a comparable delay from break onset until the start of the episodic memory task between sleep and wake conditions, by introducing a ~30 min "warum-up" period in the lab after the 90-min retention for children in the wake condition. This has now been clarified in the **Methods** section (p. 26, ll. 524-530):

"Following the ~90-min retention interval, both sleep and wake conditions included an additional ~30-min period before the memory task, either to allow recovery from sleep inertia (sleep condition) or to ensure comparable transition into the task (wake condition), respectively."

A direct comparison of retention interval duration between conditions (excluding two participants with missing timing data) revealed only a statistical trend that did not reach significance (t_{25} = 1.99, *P* = 0.058, 95% CI [-0.91, 53.21]). The distribution is shown here:

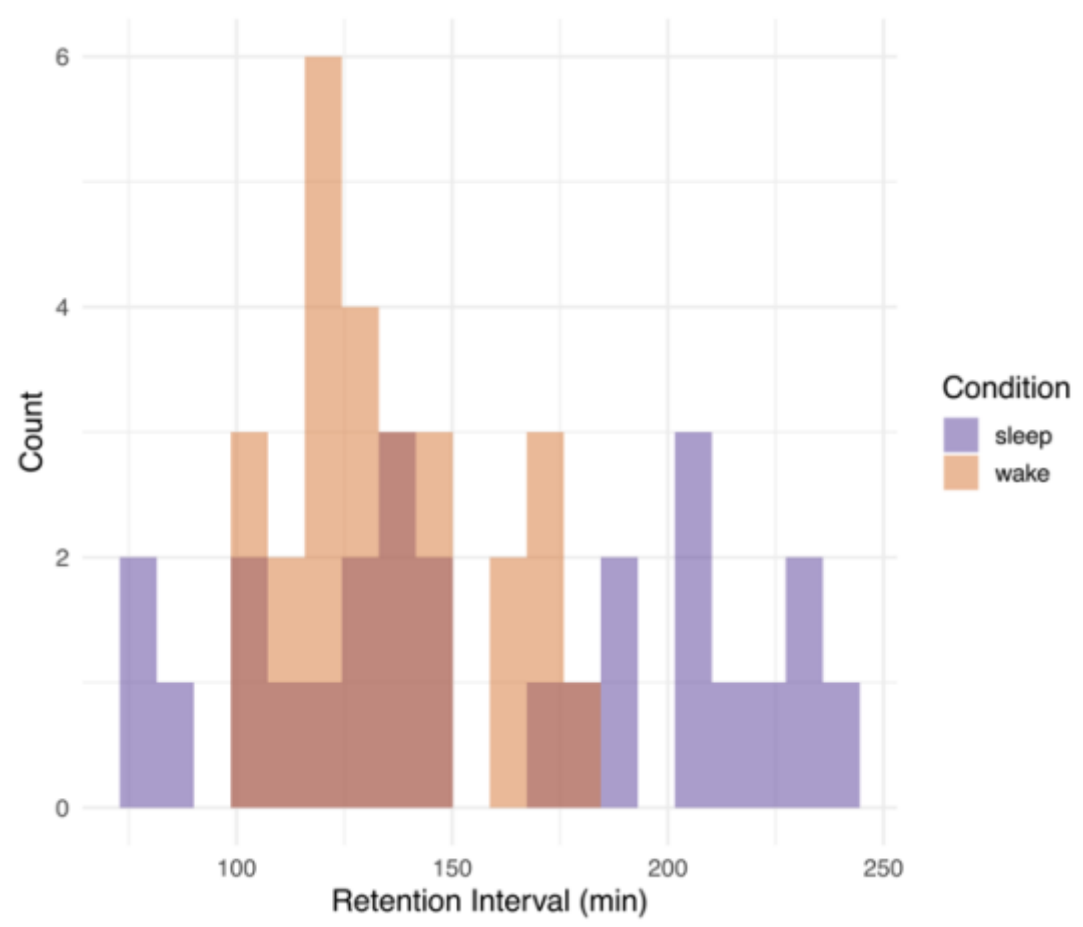

Importantly, including the rention interval as a covariate in the linear-mixed-effects models did not yield any effect of this covariate on context error rate (*β* = -0.03, SE = 0.05, *P* = 0.586, 95% CI [-0.13, 0.07]) and did not account for the variance of the effect of Sleep/Wake on context error rate which remained significant (*β* = 115.19, SE = 48.12, *P* = 0.019, 95% CI [22.99, 207.39]). Similarly, the CMI and DMI were not affected by the retention interval duration (*β* = 0.001, SE = 0.001, *P* = 0.113, 95% CI [-0.001, 0.003]). We included this information in the supplementary materials.

Supplementary Methods (p. 4, ll. 75f):

"To assess differences in retention interval duration between conditions we performed paired samples t-tests (N = 26).

Supplementary Note 1 (p. 5f, ll. 129-142):

"We performed additional control analyses to examine whether differences in retention interval duration contributed to the observed memory effects. The total break duration was numerically longer in the sleep (159.42 min) than in the wake condition (134.63 min). However, a direct comparison of the effective retention interval between conditions (excluding two participants with missing timing data) revealed only a statistical trend that did not reach significance (t_{25} = 1.99, P = 0.058; 95% CI [-0.9, 53.21]). We furthermore included retention interval duration as a covariate in the linear mixed-effects models predicting memory performance. Retention interval duration did not predict the context error rate (β = −0.03, SE = 0.05, P = 0.520, 95% CI [-0.13, 0.07]). Importantly, the effect of Sleep/Wake condition on context error rate remained significant after controlling for retention interval duration (β = 103.98, SE = 45.76, P = 0.025, 95% CI [22.99, 207.39]). Similarly, retention interval duration did not significantly predict the CMI/DMI (β = 0.001, SE = 0.001, P = 0.113, 95% CI [-0.001, 0.003]). Together, these analyses indicate that variation in retention interval duration does not account for the observed sleep-related effects on contextual memory."

We also included a section in the Discussion highlighting this limitation:

Discussion (p. 23, ll. 452-464):

"Another methodological consideration concerns differences in the total duration of the break period between the sleep and wake conditions. Although naps were not terminated prematurely and could therefore exceed 120 minutes, a ~30-min buffer period was included after both sleep and wake retention intervals to equate the effective delay before memory testing and to minimize sleep inertia effects. Additional control analyses confirmed that retention interval duration was not significantly different between conditions, did not predict memory performance, and did not account for the observed sleep-related reduction in context errors and increase in contextualized memory. Future studies may consider strictly matching retention intervals across conditions to further address potential time-dependent decay of memory representations. However, such an approach would come at the cost of either partially sleep-depriving participants or introducing systematic order effects if the sleep condition were always scheduled before the wake condition."

Comment 3:

The experimenter likely knew the condition (nap vs. wake) and gave standardized prompts, scored behaviors, sometimes opened the correct container after 2.5 min. Furthermore, parents were passively present. At age 2-3, small variations in prompting and expectations can make a big difference. Please clarify blinding, standardization, and any checks for expectancy effects, and consider adding an analysis limiting to trials resolved without experimenter intervention.

Response 3:

We thank the reviewer for highlighting this important issue. As correctly noted, the experimenter was not blinded to the conditions. This was an intentional design choice, as each child completed the entire experiment with the same experimenter they had been familiarized with during the screening session to reduce shyness, increase task engagement, and minimize distraction, thereby improving data quality.

To minimize potential expectancy or prompting effects, several precautions were taken. First, all task instructions and prompts followed a strictly standardized script, which was used identically across sleep and wake conditions. The experimenter was instructed to provide only predefined, neutral prompts and no corrective feedback during encoding or retrieval. Parents were present but instructed to remain passive and not to interact with the child during task performance. Second, all behavioral scoring was performed offline from video recordings by an independent rater who was not involved in data acquisition and was blind to the conditions. During video scoring, all verbal and non-verbal behaviors by the experimenter or parent that could potentially cue the child were explicitly coded. Trials containing such non-allowed behaviors were excluded from further analyses.

Regarding experimenter intervention during retrieval, the correct container was opened only if the child failed to locate the puppet within 2.5 minutes. This procedure was applied identically across conditions and served to prevent excessive frustration and disengagement. Importantly, retrieval outcomes were defined based on the child's first container choice, prior to any experimenter intervention.

We have added this information in more detail to the Methods section.

Methods (p. 26, ll. 531-438):

"Because of the young age of the participants, we chose that each child completed all sessions with the same experimenter to ensure familiarity and reduce stress-related variability. Consequently, the experimenter was not blinded to the child's condition. To minimize expectancy effects, all instructions and prompts during familiarization, encoding, and retrieval followed a strictly standardized script that was applied identically across sleep and wake conditions. The experimenter was trained to use only neutral, predefined verbal prompts and to refrain from providing corrective feedback or hints. Parents were present throughout the task but were instructed to remain passive and not to interact with the child."

Methods (p. 28, ll. 591-594):

"If the puppet was not located independently by the child within 2.5 min, the experimenter opened the correct container with the puppet to prevent frustration; however, performance outcomes were defined based on the child's first container choice prior to any intervention."

Methods (p. 29, ll. 512ff, 624ff):

"Due to the time-intensive nature of manual video coding, all videos were scored by a single primary coder in multiple passes who was blind to the experimental conditions. […]

The rater also coded any verbal or non-verbal actions by the experimenter or parent that could potentially cue the child. Trials containing non-allowed prompting were excluded from analysis."

Comment 4:

CMI = correct/(correct+context) and DMI = context/(context+random) are mathematically coupled. Could it be that the phrase "at the expense of decontextualized memory" reflects this coupling rather than a cognitive trade-off? Please elaborate on how the different measures

relate to each other (what increases in one imply or do not imply for the others) in the context of task success (with larger values indicating better performance) or the opposite.

Response 4:

Below is an example case highlighting how the two indices change depending on how the relative distributions of correct trials, context error rate and random error rate change with reference to the values in the first row. As illustrated in this example, redistributing performance among the underlying categories affects CMI and DMI in different ways, showing that the two indices highlight distinct and complementary aspects of the children's recall behavior, despite them being mathematically coupled:

**Scenario**	**%Correct (c)**	**%Context Error (e)**	**%Random Error (r)**	**CMI = c / (c + e)**	**DMI = e / (e + r)**	**Qualitative Pattern**
Baseline performance	25	25	50	0.50	0.33	Reference
+Correct(25), −Random(25)	50	25	25	0.67	0.50	Both increase (different magnitude)
−Correct(25), +Random(25)	0	25	75	0.00	0.25	Both decrease (different magnitude)
+Correct(25), −Context(25)	50	0	50	1.00	0.00	CMI ↑, DMI ↓ (divergent)
+Random, −Context	25	0	75	1.00	1.00	Both increase (different interpretation)

Indeed, as noted in the methods section the two memory indices are statistically dependent ($r = -.57$), due to the fact that they both share context error rate. For this reason, we did not analyze them in separate univariate models. Instead, we used a multivariate linear mixed-effects model in which both indices were included as repeated outcome measures within each participant. Importantly, this approach explicitly accounts for their non-independence by allowing correlated residuals, outcome-specific residual variances, and subject-specific random slopes for outcome. Accordingly, the model does not assume that CMI and DMI are entirely independent constructs but tests whether experimental predictors (sleep/wake condition, age, room sequence, and transition probability) relate differently to two complementary contrasts (CMI/DMI) within the same constrained response distribution (i.e. correct responses, context errors and random errors). In other words, significant effects of Sleep/Wake on CMI and DMI within this model refer to the non-correlated, i.e., uncoupled

variance of these parameters. Therefore, we believe that based on our statistical model, the phrasings like "after sleep the CMI decreased at the expense of decontextualized memory" are in principle justified. On a conceptual level, we have clarified in the revised manuscript that effects on CMI and DMI should be interpreted as differential parametrizations of the same underlying behavior, not as evidence for independent memory systems. These changes were adapted in **Fig.2e** as well as in the corresponding Results and Methods sections.

Figure 2:

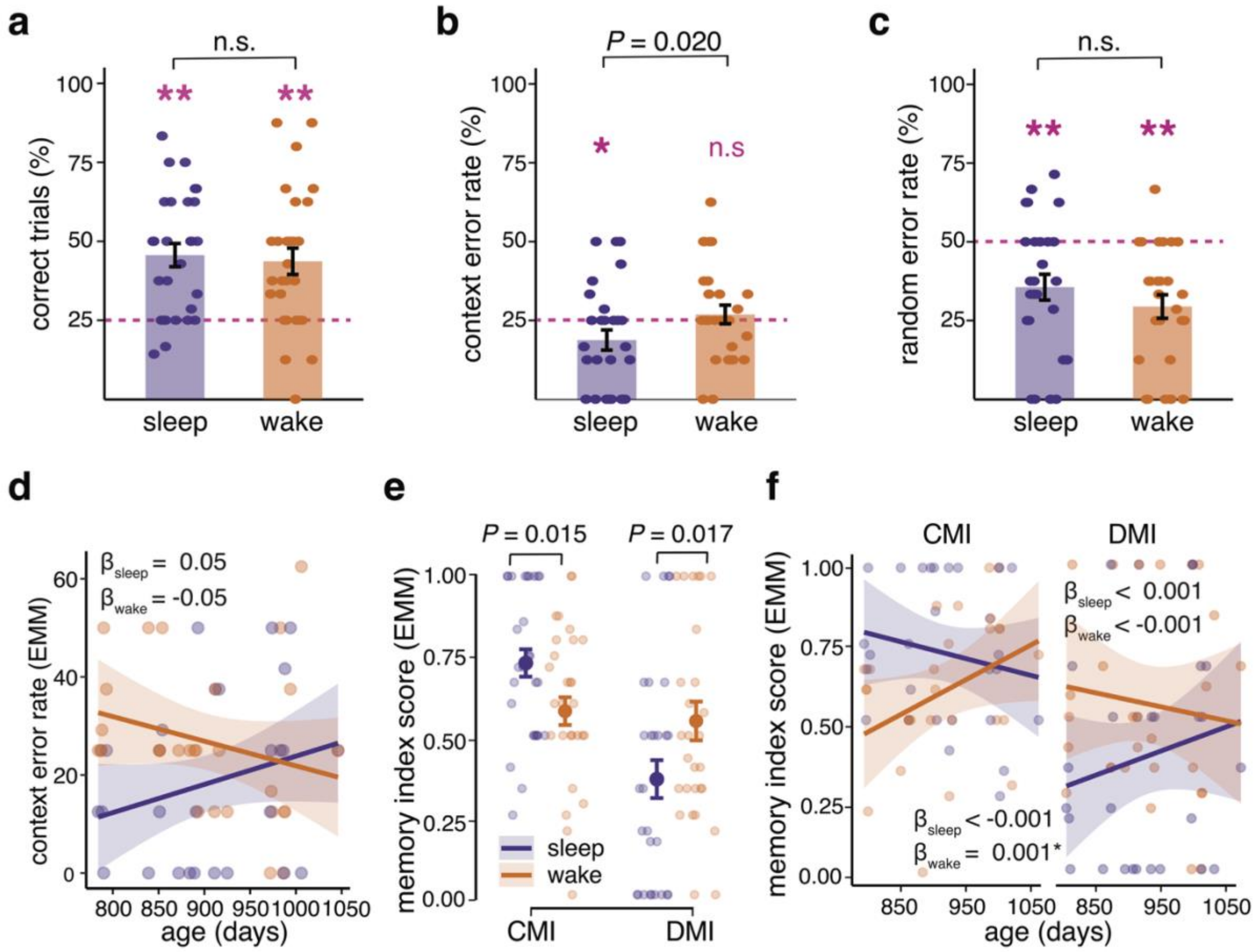

Methods (p. 30f,ll. 651-665):

"To dissociate different aspects of recall behavior, we derived two complementary indices from the same response distribution. The Contextualized Memory Index (CMI) is defined as:

$$CMI = \frac{percent\ correct\ trials}{percent\ correct\ trials\ +\ context\ error\ rate}$$

The CMI reflects the proportion of successful context–object associations out of all context-relevant responses and quantifies the child's ability to bind object memory to its spatial context. Conversely, to isolate memory for container identity independent of spatial context, we computed a Decontextualized Memory Index (DMI) as:

$$DMI = \frac{context\ error\ rate}{context\ error\ rate\ +\ random\ error\ rate}$$

By excluding trials with accurate context placement, the DMI specifically indexes container memory stripped from context-binding influences.

Both indices share the context error rate and are therefore statistically dependent. Accordingly, experimental effects on CMI and DMI are therefore interpreted as differential parametrizations of the same recall behavior."

Results (p. 9f, ll. 160-167):

"When accounting for the shared variance of CMI and DMI using multivariate linear-mixed effects model including both, the CMI and DMI, we observed an increased CMI (β = 0.15, SE = 0.07, P = 0.015, 95% CI [0.01 – 0.27]) and a decreased DMI (β = -0.15, SE = 0.07, P = 0.017, 95% CI [-0.30 – -0.01], ***Fig. 2e****) after sleep compared to wakefulness, suggesting that sleep enhances a contextualized memory, while memory for the containers is reduced after sleep. Importantly, the observed increase in CMI and decrease in DMI after sleep reflects a relative shift toward fewer context errors among all container-related decisions, consistent with more precise use of spatial context information during recall."*

Comment 5:

The GNN classifies sleep vs. wake from encoding behavior and GNNExplainer identifies the "look-into-container - container-play" transition, which is then related to performance. This approach appears to be risking circular analysis, as the same data are used to identify features and then to test their relationship with performance. Please comment on this potential issue or include an additional analysis to mitigate it.

Response 5:

We thank the Reviewer for raising this important point. We agree that circularity can arise if the same outcome variable is used both to identify predictive features and to test their association with that outcome. However, our analysis does not use memory performance at any point in training the GNN or identifying behavioral features, and therefore does not constitute a circular analysis.

First, the GNN was trained exclusively to classify Sleep vs. Wake based on the trial-specific behavior graphs during encoding, using only node features, edge features, and trial metadata that did not include the memory outcomes (i.e. correct trials, context errors, random errors, CMI/DMI). Furthermore, feature identification concerned Sleep/Wake differences only. The GNNExplainer revealed that differences in Sleep vs. Wake encoding behavior were driven predominantly by the transition from "looking into the container" to "container play." This explanatory step pertains strictly to the condition manipulation, not to memory performance. And lastly, the relationship with memory was tested in an entirely independent statistical model. After identifying the sleep-wake–sensitive transition, we examined whether this transition predicted memory performance (i.e. CMI/DMI) using separate linear mixed-effects models. Importantly, the GNN did not have access to memory performance data; thus memory performance could not influence feature identification.

Comment 6:

You state “clear signs of hippocampal immaturity”. Please specify what is meant (myelination, volume, connectivity, etc.) and summarize key findings. Please make explicit that no direct measures of hippocampal structure or function were collected here.

Response 6:

This is a important point and parts of this comment has been addressed in response to Comment 2a by Reviewer 1 (please see above). In addition, we have specified the signs of hippocampal immaturity in the **Discussion** (p. 20, ll. 376-382 & ll. 396f):

“Structural MRI shows continued volumetric growth of the hippocampus across the first two years of life[34] and protracted, subfield-specific maturation well into childhood and adolescence, with CA1, dentate gyrus and subiculum volumes continuing to change across early and middle childhood and relating to age-dependent improvements in memory[68,69]. Diffusion-based measures similarly indicate ongoing refinement of hippocampal microstructure between 4–8 years that is correlated with better episodic memory[70]. Together, these findings support the view that, at 2–3 years, the hippocampal system is still immature”.

“It is important to note that in the present study we did not collect direct measures of hippocampal structure or activity.”

Comment 7:

With central scalp EEG only (C3/Cz/C4), correlations between spindles/SO-spindle coupling and behavior are consistent with thalamo-cortical involvement but do not establish hippocampal replay or an adult-like hippocampal–neocortical dialogue. I strongly recommend to tone down mechanistic equivalence claims and discuss alternatives (e.g., predominantly thalamo-cortical consolidation at this age). The discussion appeals to the medial prefrontal cortex support for contextual abstraction, but this structure is also immature at 2-3 years. Please acknowledge this and temper the argument or cite evidence for early functional involvement despite immaturity.

Response 7:

We entirely agree. Indeed, we did not intend to infer from our findings any (adult-like) involvement of the hippocampus in sleep-dependent memory formation in our toddlers. Following the Reviewer’s suggestion, we have now rephrased the respective part of the Discussion, toning down inferences to adult-like hippocampal replay and, instead, discuss alternatives, specifically predominantly thalamo-cortical consolidation. We also now explicitly state that the medial prefrontal cortex is immature as well in toddlers. See changes in the respective **Discussion** paragraphs on p. 19f, ll. 367ff:

“[…] *In conjunction with this evidence, our findings of phase-amplitude SO-spindle coupling predicting enhanced context memory in the toddlers would agree with the concept of an active systems consolidation assuming that the consolidation process during sleep originates from hippocampal replay of contextual information during NonREM sleep [3,6,65].*

Alternatively, the sleep-dependent enhancement of context memory which we observe in our toddlers may be a consequence of a consolidation process that is restricted to the thalamo-cortical system with negligible inputs from hippocampal memory replay-related activity. This view of a predominant consolidation within the thalamo-cortical system is not unlikely given the evidence for structural and functional immaturity of the hippocampus at this early age[21,28]. […]

Accordingly, the sleep-dependent enhancement of context memory which we observe in our toddlers may alternatively be a consequence of consolidation processes within the thalamo-cortical system with negligible hippocampal inputs. It is important to note that in the present study we did not collect direct measures of hippocampal structure or activity. Thus, the question whether sleep-dependent consolidation during early life involves, like in adults, a dialogue between hippocampus and neocortex, or represents a process restricted to thalamo-cortical circuits, remains to be resolved in future research. […]

Whatever the hippocampal contribution to the consolidation process is, memory processing within the thalamo-cortical networks during sleep is thought to support the formation of more generalized and abstract representations during early development[84–86]. […] Such transfer function, i.e., to use memorized contextual information for coping with novel task situations is commonly ascribed to neocortical, specifically prefrontal cortical regions [87,88], which, notably, is also functionally immature in toddlers[50].”

To emphasize that similarities in toddlers to adult-like consolidation processes is restricted to thalamo-cotical networks, we have also changed the last sentence of the **Abstract**:

“Despite hippocampal immaturity, the sleeping toddler’s brain in thalamo-cortical networks seems to engage consolidation processes similar to those in adults to form spatial context memory for the flexible use in novel situations.”

Minor concerns

Introduction

• The opening paragraph presents SOs, spindles, and ripples in a rather condensed manner. Given the interdisciplinary audience, I recommend to briefly introduce each oscillation (frequency, generator, functional role) before discussing their coupling.

Thank you, we added the suggested information to the opening paragraph of the **Introduction** (p. 3, ll. 3-8):

“This consolidation process is thought to originate from the repeated replay of newly encoded hippocampal representations during non-rapid eye movement (NonREM) sleep, driven by ripples in the hippocampus and cortex[6] (~100 Hz). These ripples are thought to group cofiring within widespread cortical sites during time windows of neural plasticity that are defined by cortical down-to-upstate transitions (i.e. slow oscillations (SOs) with < 1Hz) and local 12–16 Hz spindles, originating from the thalamus [7–10]. The co-occurrence of ripples and spindles,

particularly when nested into the excitable SO up-state, are crucial for the long-term storage of memories within neocortical networks[11–14].”

• The background on hippocampal maturation should be expanded: what developmental markers are relevant, and how they bear on consolidation.

Despite only sparse evidence for concrete developmental markers of hippocampal maturation in humans (see Bevandic et al., 2024), we now speculated about the concrete mechanisms of successful memory formation and consolidation that may be relevant in the age range of 2-3 years and could explain the benefit of sleep (p. 3, ll. 14-21; p.3, ll. 23-25):

“Indeed, structural and functional hippocampal development follows a prolonged trajectory[23], involving processes such as myelination of neuronal axons and ongoing neurogenesis, which continue into later childhood or even adulthood [24–27]. Specifically, subregions of the hippocampus involved in the tri-synaptic pathway suggested to be crucial for forming an episodic memory engram[23], namely CA3 and dentate gyrus, develop well into the second year at least in rodents[28,29]. Recent volumetric measurements in humans corroborate these findings showing growth of CA3 and dentate gyrus even until early adulthood[30].”

“Successful memory consolidation relies on effective hippocampal-cortical communication pathways[34], including central output regions in the entorhinal cortex that develop rather late[35]”

• I suggest to clarify the scope and avoid promising an answer to regional relevance: the present design cannot disentangle hippocampal vs. cortical contributions.

We appreciate this suggestion and now stress already in the **Introduction** that our design cannot disentangle hippocampal and cortical contributions (pp. 4, ll. 52-64):

“Here, we used a spatial paradigm adapted from Newcombe et al.[47] to examine the effects of spindle-SO dynamics during sleep on spatial memory capabilities in toddlers aged between 25 and 34 months, i.e., an age when the hippocampus still shows clear signs of immaturity with spatial and episodic memory functions only starting to emerge. Importantly, the present study was not designed to disentangle the relative contributions of hippocampal versus cortical regions to memory consolidation. While the task and behavioral outcomes are widely considered to rely on hippocampal-dependent context representations, and while sleep spindle and slow-oscillation dynamics are established markers of systems-level consolidation, our design does not allow for direct inferences about the regional origin of the observed effects. Rather, our aim was to examine whether sleep at this developmental stage supports the formation of flexible context representations at the behavioral level and whether this process is associated with canonical sleep oscillations known to mediate hippocampo-neocortical communication.”

<u>Results</u>

• The attenuation of sleep benefit with age is interesting. I suggest to further elaborate on this point and potential explanations.

We were also somewhat surprised to observe an effect of age in a sample with such a narrow age range. Thus, we changed the unit of age from months to days to capture the age distribution more precisely (see the response to Reviewer 1, Comment 3 for more details). Even then, the Condition x Age interaction effect on memory contextualization remained (Fig. 2d/f). We included a paragraph in the Discussion providing potential explanations.

Discussion (pp. 18f, ll. 329-340):

"Interestingly, sleep benefits for memory contextualization were most pronounced in younger toddlers (roughly 24-30 months) considering that sleep-related reduction in context errors was attenuated with increasing age. This age range may reflect an early developmental window in which wake-based maintenance of distinct context representations is particularly fragile, rendering toddlers prone to context confusions unless sleep intervenes. This account aligns with developmental evidence that flexible contextual what–where binding shows a marked inflection around the second year but continues to refine over subsequent years, and with proposals that the transition out of naps reflects increasing hippocampal capacity to retain newly encoded information across wake [22,50,64]. In addition, age-related improvements in attentional control and emerging executive strategies may allow older toddlers to disambiguate contexts during wake by relying on online cues, reducing context errors even without sleep-dependent abstraction[50]. Together, these findings suggest that sleep confers its strongest benefit for context memory when wake-based retention of contextual information is still fragile."

• You define 10 behavioral states; please report how they were validated, inter-rater reliability, and whether rare states were pooled.

Thank you for pointing out this omission. We have now clarified the development, validation, and reliability of the behavioral coding scheme in the Methods section. The target behaviors were defined following best-practice recommendations for video-based behavioral coding as outlined in the Datavyu User Guide (PLAY Project; https://play-project.org/), in combination with task-specific information derived from the paradigm described by Newcombe et al. (2014). The coding scheme was developed in an iterative manner based on five independent pilot datasets. Starting from an initially coarse set of behaviors defined on the first dataset, the scheme was systematically refined across the remaining datasets through repeated rounds of error checking, inter-rater reliability assessment, and preliminary descriptive analyses. This process resulted in the final set of 10 behavioral states used in the main analyses.

All behaviors were defined using explicit or implicit coding criteria, which were documented in a spreadsheet-based coding manual detailing inclusion and exclusion rules, temporal boundaries, and actor assignments for each state. Due to the substantial time demands of fine-grained manual video scoring, all videos were coded by a single primary coder in several passes. Following recommended procedures, inter-rater reliability was assessed on a subset of 20% of the videos using an independent reliability coder. Agreement was quantified using Cohen's κ, computed over 2-s time windows directly within the scoring software (BORIS, v8.21.10), yielding substantial agreement ($\kappa = 0.73$).

Rare behavioral states were not pooled. This decision was motivated by the subsequent graph-neural-network analysis, which relies on preserving the full behavioral state space to

accurately capture sequential structure and transition probabilities between behaviors. Pooling infrequent states would have reduced the interpretability and granularity of the behavioral graphs.

The **Methods** section has been updated accordingly (p. 28f, ll. 598–608):

"All familiarization and encoding phases of the spatial memory task were video recorded using Axis M1065-LW IP cameras. Recordings were analyzed with BORIS software (v8.21.10). One participant was excluded due to technical failure. Behavioral scoring focused on a predefined set of 10 target behaviors (Table S1), capturing child, parent, and experimenter actions relevant to task engagement and encoding. The coding scheme was developed iteratively based on five independent pilot datasets, following established best practices for behavioral video coding (Datavyu User Guide, PLAY Project). An initial coding scheme was defined on one dataset and subsequently refined across the remaining pilot datasets through repeated rounds of error checking, inter-rater reliability assessment, and descriptive analyses, resulting in the final set of behavioral states.

All behaviors were defined using explicit coding criteria summarized in a detailed coding manual specifying behavioral definitions, temporal boundaries, and actor assignments. Behaviors were coded as either point events (occurring at a single time point) or state events (with defined onset and offset). From point events, we derived the total duration of each encoding trial, i.e. the time from "room entrance" to "room exit". Due to the time-intensive nature of manual video coding, all videos were scored by a single primary coder in multiple passes. Inter-rater reliability was assessed on a randomly selected subset of 20% of the videos using an independent reliability coder, following a first-coder/reliability-coder approach. Agreement was quantified using Cohen's κ computed over 2-s time windows, yielding substantial inter-rater reliability (κ = 0.73). "

• In Figure 3, colors for experimenter vs parent are hard to distinguish.

Based on a major comment by Reviewer 1 (see Comment 4), we decided to move panel 3a from the Tesults section into a Methods figure explaining the Graph neural network analysis step by step rather than just showing the average graph across conditions.

• Please state explicitly whether all spindle analyses were conducted separately for slow (12–13 Hz) and fast (13–16 Hz) bands.

Only spindle-SO coupling analyses were conducted for generic slow and fast spindle bands separately, not the analyses for spindles parameters because the spindle events were detected based on the individual spindle frequency peaks. Additional confusion may have arisen from the fact that we show spindle-SO phase amplitude coupling for the full 12-16Hz in panel **4c** . We changed this panel which now shows the phase-amplitude coupling for the 12-13 Hz range, the band that was used in the analysis shown in **Fig. 4d**.

Figure 4:

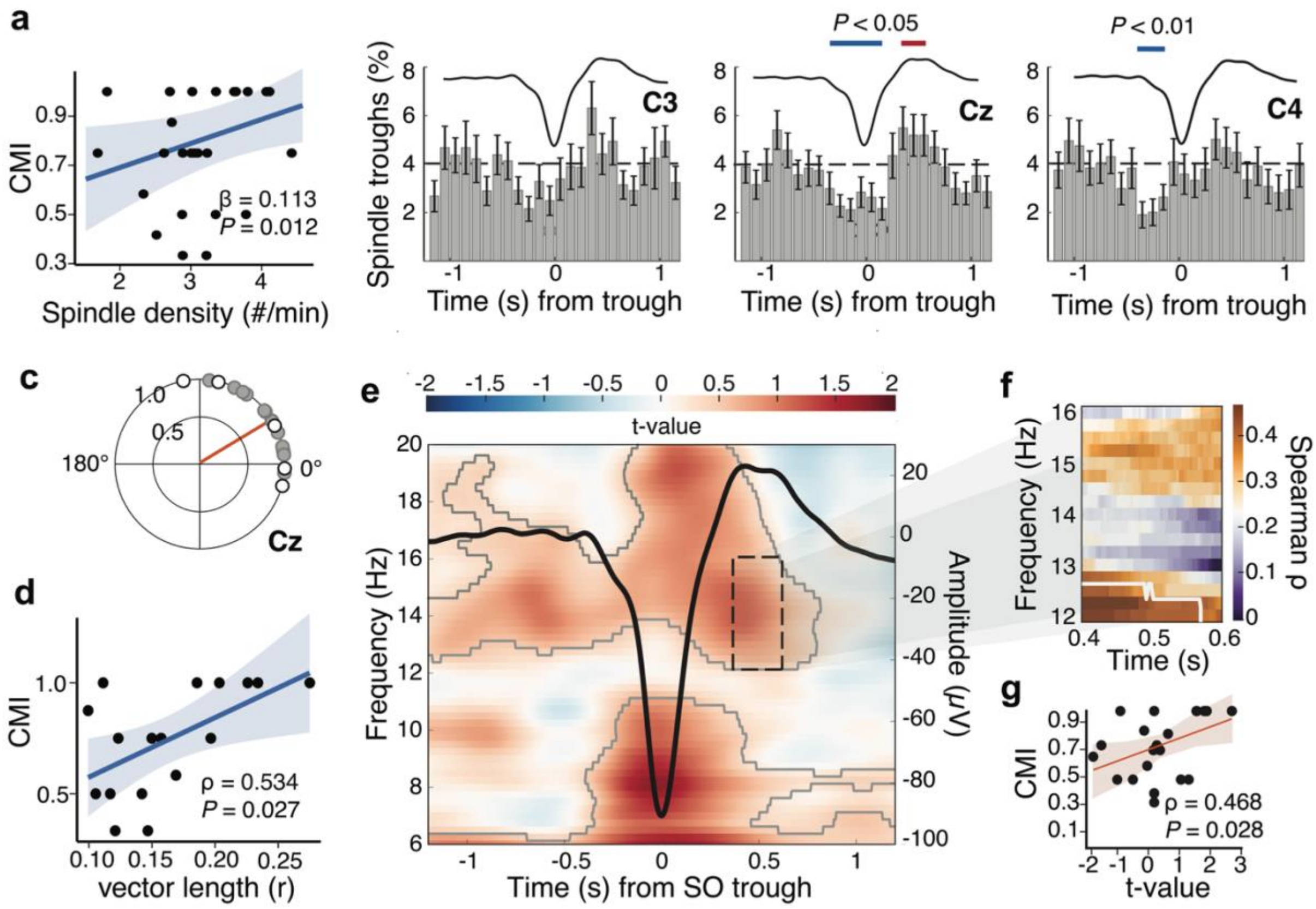

• In Figure 4, some scatter plots seem to have fewer points. I recommend adding the sample size for each analysis to every panel and clarify exclusions. I suggest to briefly define "surrogate data" in the caption.

Thank you, we added this information to the figure captions and elaborated more on the sample size in several sections of the manuscript (see response to the next comment).

<u>Methods</u>

• Please make explicit whether 28 is the final analyzed sample (after dropouts), how many were recruited initially and how the eight additional children excluded for insufficient cooperation/illness reconcile with the final sample size.

We agree that the Methods section on Participants may be misleading, To clarify:

We recruited 41 children. Out of these, five dropped out during data acquisition, four after screening and one during the first test session. Additionally, eight children were excluded post-hoc because they did not complete any trials in one of the conditions without the help of the experimenter, either due to insufficient cooperation with the experimenter (N = 7) or illness (N = 1). Thus, the final sample that was analysed consisted of 28 children. Out of these, we did not have any polysomnographic recordings for 3 children due to a lack of cooperation resulting in N = 25 for the sleep analyses from which 3 children did not have sufficient data quality over channel Cz. Since we only had three channels in total, we decided not to interpolate the data

but exclude those children for the sleep analyses (i.e., N = 22 for Cz). Lastly, 5 children were excluded for the spindle-slow oscillation phase-amplitude coupling x CMI analyses (**Fig. 4d**) because only 17 out of the 22 children had a meaningful relationship between spindle amplitude and SO phase in the 12-13 Hz range (see Rayleigh tests and filled dots in **Fig. 4c**). Additionally, video recordings were missing for one participant due to technical failure. All analyses including transition probabilities are thus based on N = 27. As stated in the previous comment, we specified the sample sizes for each analysis in the supplementary tables, the figure captions and the respective methods sections.

Participants (p. 25, ll. 491-496):

"Initially, 41 toddlers were recruited for participation in the study. Five children dropped out during data acquisition, four after the screening session and one during the first experimental condition. In addition, eight children were excluded post hoc because they did not complete any trials in at least one condition without experimenter assistance, either due to insufficient cooperation (N = 7) or illness (N = 1). Thus, the final behavioral sample consisted of 28 toddlers (29.46 ± 2.57 months; range: 25–34 months; 12 female)."

Video recordings and scoring (p. 29, l. 600):

"One participant was excluded due to technical failure ."

Sleep-EEG Recordings (p. 30, l. 643-645):

"*EEG data was acquired for all but three participants due to insufficient cooperation during electrode placement. Thus, respective sleep EEG analyses included recordings from N = 25 toddlers."*

Sleep and Behavior Analyses (p. 37, ll. 826f):

"Finally, phase-amplitude coupling (vector length) was correlated with the CMI using Spearman's ρ for those subjects with a significant Rayleigh test."

• Please specify whether EEG epochs with artifacts were rejected or interpolated, and the criteria for channel/epoch exclusion.

Thank you for the remark. We added this information to the **Methods** section (p. 32, ll. 698-702):

"Channels were rejected based on visual inspection of the power spectra. For the remaining channels, 30s-EEG epochs with artifacts labeled as movement arousals during sleep scoring were rejected from further analysis; for 3 participants channels Cz and C4, and for 4 participants channel C3 were excluded."

• Please state precisely the corrections for multiple comparisons used across analyses.

For all hypotheses-driven analyses (see Tables S5-S7) we used linear-mixed-effects models and performed multiple-comparison-corrections for all follow-up tests using Bonferroni-Holm

correction implemented in the emmeans package in R. As stated in the text. the exploratory analyses in table S4 were uncorrected for multiple comparions. Time-frequency x CMI analyes (**Fig. 4 e-g**) were 2D cluster-corrected (see Methods).

• "In models for correct trials and random errors the interaction was removed." – was this done because it yielded the most parsimonious model?

Indeed, we now provide the full scripts of the analysis in R. In these scripts the model reductions can be followed step-by-step, first printing the full models and then the reduced, most parsimonious versions (if applicable).

• Why is TST not included in Table S4?

This is a good point and we added this correlation and also the correlations for WASO to the table for completion.

• Does boldface in Tables S5 and S6 represent statistically significant results? Please specify and correct inconsistencies (e.g., Condition in S6 not bolded; Outcome×Condition in S5 bolded despite non-significance). I am confused by the numbers reported for the same analysis in the main text (page 7), as they do not match those presented in the table. If these values originate from a different analysis or model, please make this distinction clearer. Providing full model formulas throughout would be helpful.

We apologize for this mistake and corrected the tables and sections in the text accordingly. Indeed, bold face should indicate significant effects. The results reported in the main text refer to the appropriate follow-up comparisons of the effects shown in the supplementary tables (unless specified otherwise inside the brackets) to facilitate readability of the results and due to word count contraints for the main text. We added full model formulas in the **Methods** section on the statistical analysis (p.35ff).

• In Table S4, 2.94 ± 5.81 min, range 27–159.5 min appears inconsistent (that SD seems incompatible with the range). Please verify.

If we understand correctly, this comment refers to Table S2. In Table S2 we show the SEM instead of the SD. The SD was 30.74 min. We agree that it is more appropriate to report the SD for sample descrtiptives when no statistical tests are performed and therefore changed the SEM values to SD in Table S2.

References

Abney, D. H., Dale, R., Louwerse, M. M., & Kello, C. T. (2018). The bursts and lulls of multimodal interaction: Temporal distributions of behavior reveal differences between verbal and non-verbal communication. *Cognitive science*, *42*(4), 1297-1316.

Berhenke, A., Miller, A. L., Brown, E., Seifer, R., & Dickstein, S. (2011). Observed emotional and behavioral indicators of motivation predict school readiness in Head Start graduates. *Early childhood research quarterly*, *26*(4), 430-441.

Bevandić, J., Chareyron, L. J., Bachevalier, J., Cacucci, F., Genzel, L., Newcombe, N. S., ... & Ólafsdóttir, H. F. (2024). Episodic memory development: Bridging animal and human research. *Neuron*, *112*(7), 1060-1080.

Blankenship, S. L., Redcay, E., Dougherty, L. R., & Riggins, T. (2017). Development of hippocampal functional connectivity during childhood. *Human brain mapping, 38*(1), 182–201. https://doi.org/10.1002/hbm.23353

Friedrich, M., Mölle, M., Friederici, A. D., & Born, J. (2019). The reciprocal relation between sleep and memory in infancy: Memory-dependent adjustment of sleep spindles and spindle-dependent improvement of memories. *Developmental science*, *22*(2), e12743. https://doi.org/10.1111/desc.12743

Friedrich, M., Mölle, M., Born, J., & Friederici, A. D. (2022). Memory for nonadjacent dependencies in the first year of life and its relation to sleep. *Nature Communications*, *13*(1), 7896.

Keresztes, A., Bender, A. R., Bodammer, N. C., Lindenberger, U., Shing, Y. L., & Werkle Bergner, M. (2017). Hippocampal maturity promotes memory distinctiveness in childhood and adolescence. *Proceedings of the National Academy of Sciences of the United States of America*, *114*(34), 9212–9217. https://doi.org/10.1073/pnas.1710654114

Kurz, E. M., Bastian, L., Mölle, M., Born, J., & Friedrich, M. (2024). Development of slow oscillation–spindle coupling from infancy to toddlerhood. *Sleep Advances*, *5*(1),

zpae084.

Mölle, M., Marshall, L., Gais, S., & Born, J. (2002). Grouping of spindle activity during slow oscillations in human non-rapid eye movement sleep. *The Journal of neuroscience : the official journal of the Society for Neuroscience*, *22*(24), 10941–10947. https://doi.org/10.1523/JNEUROSCI.22-24-10941.2002

Randler, C., Faßl, C., & Kalb, N. (2017). From Lark to Owl: developmental changes in morningness-eveningness from new-borns to early adulthood. *Scientific reports*, *7*(1), 45874.

Riggins, T., Geng, F., Blankenship, S. L., & Redcay, E. (2016). Hippocampal functional connectivity and episodic memory in early childhood. *Developmental cognitive neuroscience*, *19*, 58–69. https://doi.org/10.1016/j.dcn.2016.02.002

Tamis-LeMonda, C. S., & Masek, L. R. (2023). Embodied and embedded learning: Child, caregiver, and context. *Current Directions in Psychological Science*, *32*(5), 369-378.

Xu, T. L., De Barbaro, K., Abney, D. H., & Cox, R. F. (2020). Finding structure in time: Visualizing and analyzing behavioral time series. *Frontiers in Psychology*, *11*, 1457.

Response to Reviewers

We thank the Reviewers for the constructive assessment of our revised manuscript and are very pleased that the major concerns raised in the first round are now considered adequately addressed. Considering the remaining comments, we have further moderated the interpretation of our findings, included model fits, and changed formulations to increase transparency regarding sample size and statistical analyses. We believe that these changes strengthen the manuscript without altering its main conclusions. All changes are marked in red in the revised version of the manuscript.

Comment 1

In the abstract: "the sleeping toddler's brain seems to engage consolidation processes similar to those in adults"; given the acknowledged absence of direct hippocampal measures, this wording may still overstate mechanistic insights.

Response 1

We have revised the respective sentence to emphasize the similarity of the observed sleep-related behavioral and thalamo-cortical EEG signatures to mechanisms implicated in adult memory consolidation, without implying hippocampal involvement:

"Here, we show that, despite hippocampal immaturity, memory effects in the sleeping toddler's brain are accompanied by thalamo-cortical oscillatory dynamics that are also implicated in adult memory consolidation."

Comment 2

On page 3, the phrasing "including central output regions in the entorhinal cortex that develop rather late" could be more precise. "Rather late" is somewhat colloquial and vague.

Response 2

We have replaced the original wording with "*which show prolonged postnatal maturation in some subdivisions, as demonstrated in non-human primates*" (p.3, l.25f), to be more specific. We refrained from estimating a concrete age because we did not find any work providing direct evidence in humans.

Comment 3

While I appreciate the practical challenges of this population, several key analyses rely on relatively small subsamples (e.g., N = 22 for EEG analyses, N = 27 for transition probability analyses, and N = 17 for coupling analyses). I recommend to further acknowledge the implications of limited statistical power and stability of effect estimates in the Discussion.

Response 3

We have now added the following statement to the Discussion (p.10, ll.356ff):

"… are the first to show that beyond spindle density [15,36], also the precise phase-coupling of spindle amplitudes to the SO-upstate predicts better contextual memory. Although this finding was consistent across analyses, it should be noted that our EEG-coupling analyses were performed on a smaller subsample, which limits the precision and stability of the corresponding

effect estimates. Future replication in larger samples will be important to establish the robustness of the EEG-behavior relationships."

Comment 4

Relatedly, the exclusion criterion for the coupling analysis would benefit from additional justification. The response states that "5 children were excluded … because only 17 out of the 22 children had a meaningful relationship between spindle amplitude and SO phase…". Please clarify more explicitly why this exclusion is methodologically necessary and whether this criterion was defined a priori.

Response 4

We have now clarified that data from participants with non-significant coupling (in terms of Rayleigh test) were not included, because in these cases the resulting coupling measure does not provide a reliable estimate of the phenomenon of interest and would thus not be interpretable in the subsequent correlation analysis. This approach was defined a priori as we had routinely used it previously (Bastian et al., 2022, doi: 10.1002/hbm.25893, Kurz et al., 2024, doi:10.1093/sleepadvances/zpae084; p. 21, ll. 834ff):

"Finally, phase-amplitude coupling (vector length) was correlated with the CMI using Spearman's ρ for those subjects with a significant Rayleigh test. Following previously used procedures[14,40], we included only participants who showed a deviation from a uniform distribution of spindle amplitudes across SO phase to ensure that we could obtain a reliable estimate of the relationship between phase-amplitude coupling and behavior."

Comment 5

Given the relatively small sample size, I wonder whether some of the mixed-effects models may be overparameterized. It would strengthen the manuscript to provide additional information on model performance and explained variance (e.g., marginal/conditional R^2 values where appropriate).

Response 5

Our models were reduced to the most parsimonious structure using the Akaike Information Criterion, and random effects were restricted to structures supported by the repeated-measures design. We have now additionally quantified model fit by reporting marginal and conditional R^2 values for the principal mixed-effects models in the Supplementary Tables S5 – S7.

Comment 6

The response states that the number of independent statistical tests was "<15". For transparency, I suggest explicitly reporting the exact number in the manuscript itself.

Response 6

We have now provided the exact number of independent statistical tests considered in our multiple-testing framework (p. 19, ll.767ff):

"The statistical analysis comprised 11 hypothesis-driven models and 3 exploratory analyses, including a graph-based behavioral analysis, a mediation analysis and macro-sleep architecture correlations with behavior."

Comment 7

While the Discussion has been substantially improved and appropriately toned down in many places, a few statements throughout the manuscript still appear somewhat stronger than warranted by the present data and could benefit from more cautious phrasing.

Response 7

We appreciate this comment and have made several targeted changes throughout the Discussion. Please find changes in wording regarding "abstracted/generalized" directly in the manuscript file (marked in red). Additionally, we have changed the following sentences,

(i) to highlight the exploratory nature of the GNN analysis:

- "*In an exploratory graph-based analysis, the post-familiarization nap was moreover associated with a change in the toddler's behavior during the encoding ("hide") phase of the hide-and-seek task such that…* " (p. 9, ll. 273ff)
- Where this finding is discussed in more detail we note that: "*Because this behavioral analysis was exploratory and achieved only modest classification accuracy (65.79%), we regard this interpretation as tentative"* (p. 12, ll. 421ff)

(ii) to more cautiously express the aims of our experimental design, namely that the design "*was intended to target sleep effects on context memory rather than on specific context–event binding, although, as discussed below, an additional influence of sleep on subsequent encoding cannot be fully excluded*" (p. 10, ll. 316ff)

and (iii) refined the terminology concerning "persistent context memory". The respective ,passage now states that: "*a context representation that persists across a retention interval of more than 1.5 h in human toddlers",* followed by: *"It should be emphasized that this persistence pertains to the contextual representation established during familiarization*" (p. 9, ll. 298ff).
